# Size-Dependent Surface Evolution Controls Luminescence in Ln^3+^-Doped Nanocrystals

**DOI:** 10.1021/acsnano.6c11081

**Published:** 2026-10-23

**Authors:** Rui Shi, Guillaume Bousrez, Ramkumar Venkatachalam, Anja-Verena Mudring

**Affiliations:** † Intelligent Advanced Materials, Department of Biological and Chemical Engineering and iNANO, 1006 Aarhus University, Aarhus C 8000, Denmark; ‡ Department of Physics, 8075 Umeå University, 901 87 Umeå, Sweden

**Keywords:** surface evolution, nanocrystals, luminescence, lanthanide, lattice distortion

## Abstract

Lanthanide-doped luminescent nanocrystals (NCs) hold significant promise for bioimaging, theranostics, catalysis, sensing, lighting, and displays, motivating an in-depth understanding of their optical properties. This work reveals the profound, yet widely disregarded, impact of surface structure changes on the luminescence of growing NCs through a model study of Eu^3+^ surface-exchanged Sr_2_YF_7_ NCs. Contrary to expectation, a stark drop in luminescence intensity exceeding several orders of magnitude was observed as the particle size increased. Atomic-resolution electron microscopy imaging shows that the NCs evolve from spheroidal to cuboidal morphologies during growth, with a shift in preferentially exposed crystal facets from {111} to {200} that renders lattice distortion less favorable. High-resolution optical spectroscopy probing surface Eu^3+^, together with energy-dispersive X-ray spectroscopy and reaction kinetics study, reveals a high extent of Eu^3+^ exchange in the small spheroidal NCs with lattice distortion extending far from the surface into the interior. This starkly contrasts the situation in the large cuboidal NCs, where the extent of Eu^3+^ residing in the surface region is severely limited, thereby causing the dramatic drop in luminescence. These findings demonstrate surface structure control as a powerful strategy for tailoring the luminescence properties of Ln^3+^-doped NCs and other nanomaterials for advanced applications.

## Graphical abstract

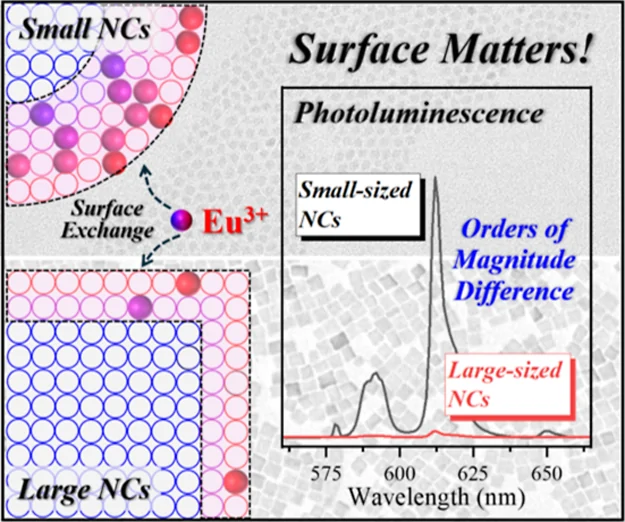

Ln^3+^-doped (Ln = lanthanide) optical materials,^1,2^ with their luminescence stemming from the electronic transitions between the Ln^3+^ 4*f* energy levels,^2–4^ have attracted tremendous attention. These intrinsic optical features,^5^ which are grounded in the well-shielded 4*f* orbitals of Ln^3+^ ions and unattainable from other materials, render this class of materials particularly promising to meet the growing demands in advanced optical nanotechnologies.^6,7^

However, the decrease in size of materials to the nanometer regime alters their properties. Although Ln^3+^-doped nanomaterials, due to their unique electronic configurations, are not affected by quantum confinement,^8^ their luminescence is notably different from their bulk counterparts.^9^ It is generally believed that the drastic rise in the surface-to-volume ratio of NCs with decreasing size^10^ is responsible for the deviation in properties, many of which are stunning,^11^ even puzzling.^12,13^ Commonly, the influence of the increased surface area of Ln^3+^-doped NCs on their luminescence is considered to be detrimental, due to the formation of surface defects and the absorption of moisture or solvent molecules that can evoke vibronic quenching.^13^ Still, there are experimental findings that cannot be explained by these notions and even appear to be contradictory.^14,15^ This necessitates a more thorough investigation of the exact implications of size reduction of these materials to the nanoscale. A widely disregarded aspect is that the surface of nanomaterials changes with varying sizes, not only in area but also in crystal structure, thereby altering the photophysical properties compared to the ideal bulk.^16^ Properly addressing this aspect will not only provide an in-depth understanding of the luminescence of Ln^3+^-doped NCs, but also allow for luminescence manipulation for target applications through surface engineering.

Deciphering this puzzle requires well-designed experiments. For probing subtle structural changes at the surface of NCs as their sizes vary, optical spectroscopy of Eu^3+^ is particularly suitable, as the characteristics of the electronic transitions between the Eu^3+^ 4*f* energy levels are directly correlated to its site symmetry (Figure 1a).^17–19^ Of equal importance is the selection of an appropriate host material, where any structural change induces a clear spectral change.^20^ Sr_2_YF_7_ (abbreviated as SYF) fulfills this demand with its crystal structure. The structure of SYF can be derived from the fluorite-type structure (CaF_2_, with *Fm3̅m* space group), where Sr^2+^ and Y^3+^ occupy the Ca^2+^ position (Wyckoff position 4*a*, site symmetry *m3̅m*) (Figure 1a). For each Y^3+^ introduced, a charge compensation is needed, which is achieved through interstitial F^–^. This leads to a symmetry distortion from *Fm3̅m* to *I*4/*m*,^21^ but leaves Y^3+^ in a highly symmetric coordination. In contrast, the cationic sites at the surface of NCs are heavily distorted from the ideal symmetry (Figure 1a). We recently demonstrated an isovalent replacement of Y^3+^ sites by Eu^3+^ in the SYF NCs and observed a noticeable difference in the spectroscopic features of Eu^3+^ ions residing at the surface or in the NC interior.^22^ This enables us to track any structural changes that are associated with the particle surface (and also the particle size) through the change in spectroscopy. For these reasons, we employ the Eu^3+^ surface-exchanged SYF NCs to unveil the close correlation between the particle size-dependent luminescence of NCs and surface structure evolution proceeding during NC growth.

**Figure 1:**
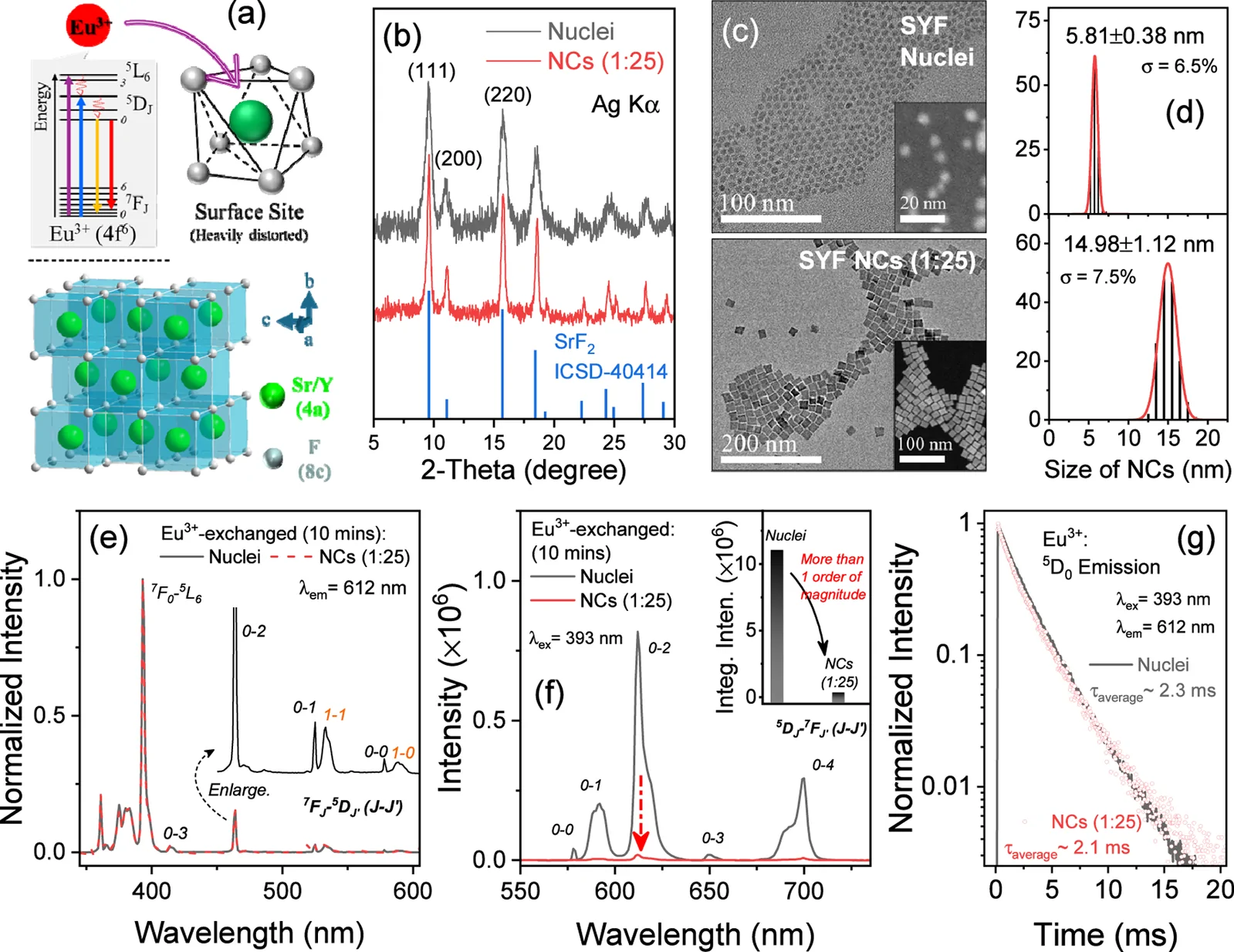
(**a**) Energy level diagram of Eu^3+^ (the 4*f* levels termed ^*2S+1*^ *L*_*J*_, where *S*, *L*, and *J* represent the spin, orbital, and total angular momentum quantum numbers, respectively) and visualization of the SYF crystal structure and the distorted coordination environment of the cationic sites at the particle surface (figuratively). (**b**) PXRD patterns of representative host SYF nuclei and NCs (1:25), along with the standard XRD pattern of SrF_2_. (**c**) TEM and wide-field STEM-HAADF imaging of the SYF nuclei and NCs (1:25) of ∼5.8 and 15 nm, respectively, and (**d**) the corresponding particle size distributions. (**e**) Normalized excitation spectra of the Eu^3+^-exchanged (10 min of exchange) NCs of different sizes by monitoring the 612 nm Eu^3+^ ^5^D_0_–^7^F_2_ emission, with magnification of the representative spectrum in the range of 450–605 nm provided in the inset, as well as the terms of Eu^3+^ 4*f*–*4f* transitions indicated. (**f**) Emission spectra of corresponding samples upon the excitation of the Eu^3+^ ^7^F_0_–^5^L_6_ transition (393 nm), with the integral luminescence intensities of samples given in the inset. (**g**) Decay dynamics and the average lifetimes of the Eu^3+^ ^5^D_0_–^7^F_2_ luminescence (612 nm) in the representative Eu^3+^-exchanged NCs of different sizes upon 393 nm excitation.

## Results and Discussion

Monodispersed ∼5.8 nm-sized SYF nuclei with uniform spheroidal morphology were produced by thermally decomposing appropriate amounts of strontium and yttrium trifluoroacetates in a mixture of 1-octadecene (ODE), oleic acid (OA), and oleylamine (OM) at 300 °C for 30 min (see METHODS for the experimental details).^22,23^ A layer-by-layer growth of SYF NCs was achieved by hot-injection of respective amounts of the trifluoroacetate precursors similar to previously described protocols.^24,25^

Powder X-ray diffraction (PXRD) confirms SYF crystallizing similar to SrF_2_. The diffraction peaks in the patterns of NCs sharpen as the particle size increases (Figure 1b). Transmission electron microscopy (TEM) and wide-field scanning transmission electron microscopy high-angle annular dark-field (STEM–HAADF) imaging of NCs (1:25) (the value in the parentheses denotes the nominal ratio in molar amounts of the nuclei seed and the precursors used for crystal growth) shows a spherical-to-cuboidal change in NC morphology (Figure 1c) as the precursor hot-injection proceeds, as the particle size reaches ∼15 nm (Figure 1d). The appearance of high-frequency vibrational modes of organic groups in the infrared spectra of NCs of varying sizes (Figure S1a) indicates surface binding of the long-chain oleic surfactant used during material synthesis, as also evidenced by the thermogravimetric analysis of representative NCs (1:25) (Figure S1b).

Hot-injection of Eu­(III) oleate into the reaction medium containing suspended SYF NCs allows for Eu^3+^ exchange at the NC surface (see the experimental details in METHODS), with no significant alterations in size (within the standard deviation of measurement) and morphology (Figure S2), as we detailed previously.^22^ This now allows for probing surface structure changes using Eu^3+^ spectroscopy, as it is commonly known from studies correlating the spectral features of Eu^3+^ with its local crystallographic coordination environment.^17^ In general, the intraconfigurational 4*f*–4*f* electronic transitions of Eu^3+^ are strongly restricted by the Laporte rule because the initial and final states share the same orbital parity. When an isolated Eu^3+^ resides in a perfectly centrosymmetric site, only weak magnetic–dipole transitions remain active, while pure electric–dipole transitions are completely forbidden. Nevertheless, these restrictions are relaxed when Eu^3+^ is embedded in a distorted coordination environment. The noncentrosymmetric crystal field introduces the opposite-parity components (such as 5*d* orbitals) into the 4*f* configurations, breaking the Laporte rule of parity restriction and driving a dramatic increase in intensity for “forced” electric–dipole transitions.^26^

The normalized excitation spectra of representative Eu^3+^-exchanged NCs of varying sizes collected by monitoring the characteristic Eu^3+^ ^5^D_0_–^7^F_2_ “forced” electric–dipole transition (Δ*J* = 2) at 612 nm show the dominance of the hypersensitive ^7^F_0_–^5^D_2_ excitation (Δ*J* = 2) of Eu^3+^ over its ^7^F_0_–^5^D_1_ magnetic–dipole transition (Δ*J* = 1), with the intense ^7^F_0_–^5^L_6_ (393 nm) and feeble ^7^F_0_–^5^D_3_ lines observed (Figure 1e). Signals associated with the ^7^F_1_–^5^D_1_, ^7^F_1_–^5^D_0_, and ^7^F_0_–^5^D_0_ transitions of Eu^3+^ were also observed (Figure 1e, inset), with the hypersensitive transition emerging, which is expected as the transition selection rule is relaxed.^26,27^

Upon excitation of the Eu^3+^ ^7^F_0_–^5^L_6_ transition at 393 nm, the characteristic emissions of Eu^3+^ with its ^5^D_0_–^7^F_2_ transition being dominant over the ^5^D_0_–^7^F_1,4_ transitions appear (Figures 1f and S3), with a sole ^5^D_0_–^7^F_0_ line seen at around 579 nm, in agreement with our previous observations.^22^ These findings clearly show that Eu^3+^ resides exclusively at the surface of these NCs, with its site symmetry strongly distorted (such as toward *C*_*1*_ or *C*_*2*_ symmetry^27^) as demonstrated previously^22^ through explicit analyses of spectral fine structures of Eu^3+^. The formation of Eu^3+^ homogeneously alloyed NCs can be fully excluded.^22^

Notably, there is a sharp drop of more than 1 order of magnitude in the luminescence intensity of the Eu^3+^-exchanged NCs as the particle size increases (Figure 1f, inset). This difference is also visible upon excitation of the Eu^3+^ ^7^F_0_–^5^D_2_ transition at 463.5 nm (Figure S4a), which preferentially excites the Eu^3+^ residing at the outer surface of NCs with a heavily distorted local coordination as evidenced by the relaxed selection rule.^27^ As the luminescence intensity of Eu^3+^-exchanged NCs of varying sizes was measured under identical experimental conditions, any influence from experimental variations can be excluded. Also, this stark difference cannot be grounded in a mere 2.57-fold reduction in the overall surface area of the material evoked by an increase of the average NC size from 5.8 to 15 nm (this would correspond to estimated surface areas (A) of 105.68 nm^2^ (single smaller spheroidal particle) vs. 1350 nm^2^ (single larger cuboidal particle), volumes (V) of 102.16 nm^3^ vs 3375 nm^3^, and A/V ratios of 1.03 vs. 0.4, respectively).

Similar decay dynamics and the average lifetimes of the Eu^3+^ ^5^D_0_ luminescence (Figure 1g, ∼2.3 ms for the case of nuclei; ∼2.1 ms for NCs (1:25)) were collected regardless of the excitation wavelength (Figure S4b). In addition, the similar photoluminescence quantum yields (Figure S5) upon 393 nm excitation (∼27% in both cases) show that no additional de-excitation relaxation channel emerges as the particle size varies. Thus, we can rule out these effects on the sharp drop in luminescence intensity of the Eu^3+^-exchanged NCs grown in size.

Experiments were designed to explore the nature behind this stark drop in luminescence (See Figure 2a; further experimental details can be found in METHODS). Notable luminescence from Eu^3+^ residing at the surface of SYF nuclei is detected immediately after 1 min of exchange (Figure 2b) upon 393 nm excitation for 5.8 nm-sized NCs. The spectral features of Eu^3+^ remain largely unchanged as the exchange is extended to 20 min (Figure S6), with the luminescence intensities of the samples increasing toward saturation (Figure 2c). In turn, there is a gradual decrease in luminescence intensity from Eu^3+^ remaining in the supernatant liquid as the exchange progresses (Figure 2d), with spectral features (Figure S7) consistent with those observed for Eu^3+^ in the Eu­(III) oleate solution used for exchange.^22^

**Figure 2:**
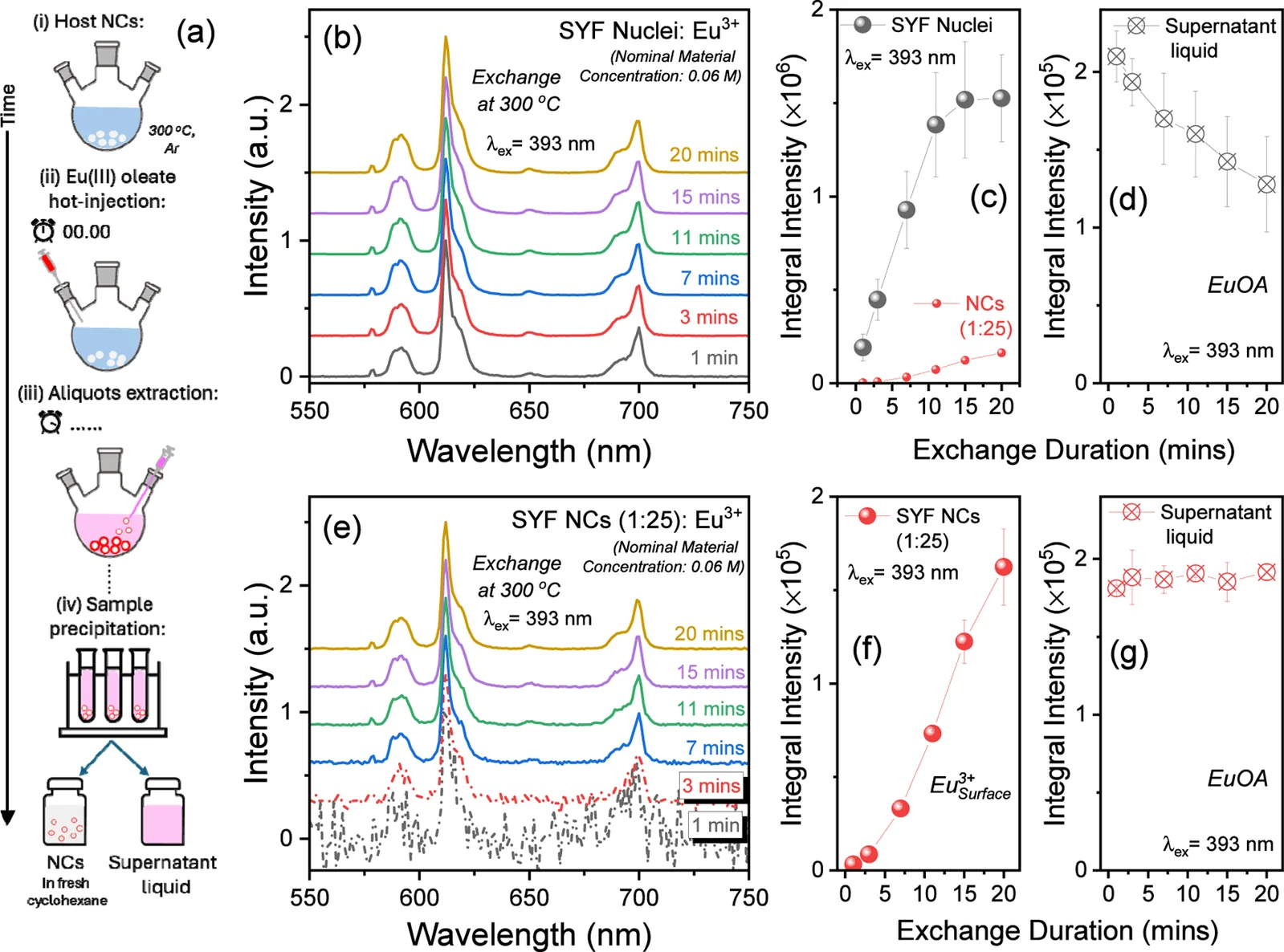
(**a**) Experimental workflow for Eu^3+^ exchange at the surface of SYF NCs, as well as procedures of sampling and separation. *i*. Neat host NCs of different sizes dispersed in the medium of OA and ODE at high temperatures with continuous Ar flow; *ii*. Hot-injection of Eu­(III) oleate dispersed in ODE to trigger the exchange of Eu^3+^; *iii*. An aliquot extraction of an equal quantity from the reaction medium with a certain period of exchange. The time reference of 0 min is set as the moment of the Eu­(III) source injection. *iv*. Precipitation of the Eu^3+^-exchanged NCs by using a mixed solvent of ethanol and hexane, with the NCs redispersed into fresh cyclohexane and the supernatant liquids collected for spectral measurements. (**b**) Comparison of the normalized emission spectra of the Eu^3+^-exchanged SYF nuclei collected with varying exchange durations (with a nominal material concentration of 0.06 M), and (**c**) the corresponding exchange duration-dependent luminescence intensity of specimens and (**d**) the supernatant liquids upon 393 nm excitation. (**e**) Normalized emission spectra of the representative Eu^3+^-exchanged NCs (1:25) collected with varying exchange durations (nominal material concentration: 0.06 M), and (**f**) the corresponding exchange duration-dependent luminescence intensity of specimens and (**g**) the supernatant liquids upon 393 nm excitation. Three sets of controlled experiments were conducted independently under identical conditions, and the data deviations obtained are presented as error bars.

In contrast, luminescence from the large-sized Eu^3+^-exchanged NCs (1:25) can only be clearly detected after 7 min of *t*_Exchange_ (Figure 2e), with the exchange temperature (*T*_Exchange_) of 300 °C. A gradual increase in the luminescence intensity of the Eu^3+^-exchanged NCs (1:25) can be witnessed when extending the *t*_Exchange_ from 7 to 20 min (Figure 2f). Meanwhile, the overall luminescence of the Eu^3+^-containing supernatant liquid does not vary much over the extended reaction period (Figure 2g), in contrast to what has been observed when employing the small-sized nuclei as the starting material.

Again, there is about 1 order of magnitude difference in the luminescence intensity of Eu^3+^-exchanged nuclei compared to that of the NCs (1:25) after 20 min of *t*_Exchange_ (Figure 2c). From this, it can be concluded that Eu^3+^ exchange is more effective at the surface of the smaller-sized nuclei compared to the larger ones. A large portion of Eu^3+^ has been exchanged at the nuclei surface in a short period, and it saturates rapidly as the exchange progresses. In comparison, the majority of Eu^3+^ remains in the reaction medium for the large NCs (1:25).

The reaction kinetics of Eu^3+^ exchange at the surface of SYF nuclei and NCs (1:25) were further studied by varying experimental conditions of *T*_Exchange_ and *t*_Exchange_ (Figure S8). While the activation of Eu^3+^ exchange at the surface of large NCs (1:25) demands high *T*_Exchange_ and long *t*_Exchange_, Eu^3+^ exchange at the nuclei surface can be initiated at rather mild conditions of low *T*_Exchange_, even at 255 °C, and 1 min of *t*_Exchange_. A striking demonstration of this difference is that lowering *T*_Exchange_ slightly from 300 to 285 °C results in a more than 2 orders of magnitude drop in the luminescence intensity of Eu^3+^-exchanged NCs (1:25) compared to that of the nuclei. These findings evidence a strong correlation between Eu^3+^ exchange and the surface features of SYF NCs of varying sizes.

The change in surface structure of SYF NCs grown in size can be interrogated through EM imaging analysis^28^ (Figure 3a–d). Along with the change in NC morphology from spheroidal (nuclei and NCs (1:3)) to subhedral cuboidal (NCs (1:5)) to cuboidal (NCs (1:25)),^29^ there is a unique particle size-dependent shift in the feature of lattice fringes visible on NCs. Statistical analysis of the proportion of NCs with different features in lattice fringes (Figure 3a.3.–d.3.) shows that most small-sized nuclei exhibit lattice fringes attributed to the {111} set of crystal planes, while different lattice fringes, mainly corresponding to the {200} set of planes, are seen for the large-sized NCs (1:25).

**Figure 3:**
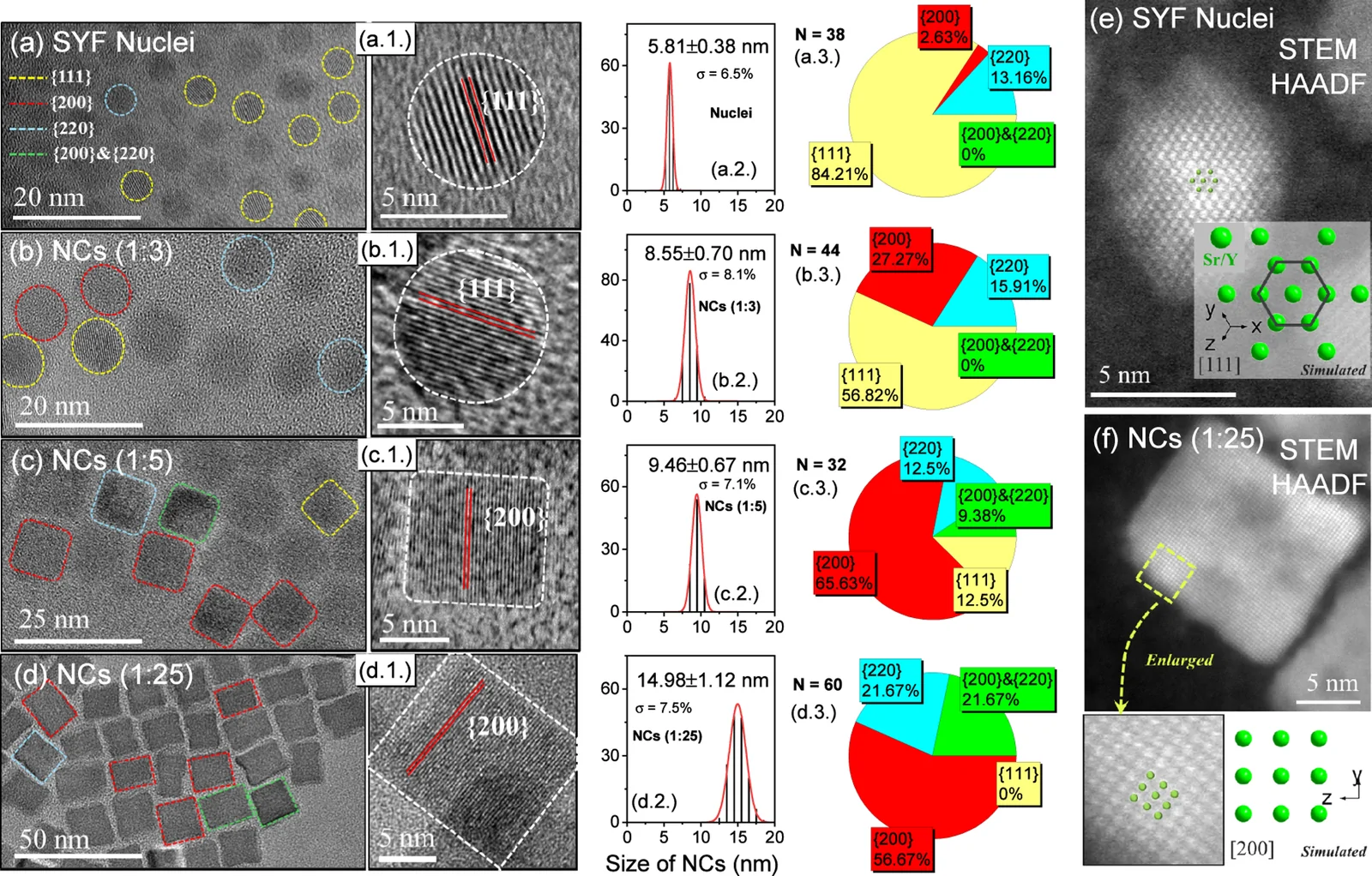
TEM imaging analysis of SYF NCs with different sizes, ranging from (**a**) nuclei, (**b**) NCs (1:3), (**c**) NCs (1:5), to (**d**) NCs (1:25). (a.1.–d.1.) Particles with clear lattice fringes (owing to the phase shift originating from Bragg scattering) observed in the cropped TEM images (full-sized images given as Figures S9–S12) are outlined in different colors depending on the sets of crystal planes associated with the lattice fringes belonging to the following set of planes {111} in yellow, {200} in red, {220} in blue, and {200} &{220} in green, assuming a *Fm3̅m* symmetry for SYF. Insets provide high-resolution TEM images of representative NCs, with clear lattice fringes observed for each sample, along with the corresponding sets of crystal planes denoted. (a.2.-d.2.) Size distributions of NCs, and (a.3.-d.3.) the plots denoting the proportion of NCs with the lattice fringes attributed to various sets of crystal planes using the same color codes, where the total number of NCs with clear lattice fringes observable for analysis is given by N, respectively. (**e**) Atomic-resolution STEM-HAADF imaging of an individual small SYF nucleus and (**f**) large NC (1:25), resolving the atom arrangement of their exposed crystal facets. The simulated Sr/Y arrangements observed along different crystal zone axes are presented in the insets, respectively, assuming a *Fm3̅m* symmetry for SYF crystal structure.

Atomic-resolution STEM–HAADF imaging of an individual nucleus and NC (1:25) (Figure 3e,f) further resolves the precise cation arrangements of their exposed crystal facets. Comparisons with the simulated Sr/Y arrangements along different crystal zone axes of SYF lattice assuming a *Fm3̅m* symmetry (Insets of Figure 3e,f), confirm the small spheroidal nuclei with {111} crystal facets exposed, whereas the large cuboidal NCs (1:25) expose {200} facets. These results manifest surface structure evolution during NC growth.

The {111} set of planes associated with the exposed facets of nuclei has a larger interplanar spacing of ∼3.34 Å compared to the NCs (1:25) with the ∼2.90 Å spacing of the {200} planes. A loose stacking of crystal planes allows for easier lattice distortion. This agrees with a rapid exchange as seen in the small nuclei where the surface is significantly distorted from the ideal that leads to a low energy barrier for exchange, in stark contrast to the large cuboidal NCs. Analysis of energy-dispersive X-ray spectroscopy of Eu^3+^-exchanged NCs (Figures S13–S16) further reveals a sharp contrast in the extent of Eu^3+^ substitution (around 1 order of magnitude difference) at the surface of NCs grown in size.

Detailed spectroscopic analysis of Eu^3+^ in the NCs of varying sizes allows for deciphering this surface structure evolution. The comparison of the excitation/emission wavelength-selected steady-state spectra (Figure S17) of the Eu^3+^-exchanged (20 min) nuclei and NCs (1:25) already visualizes the differences, especially in the normalized emission spectra of samples (Figure S17c,d), where the relative intensity of the Eu^3+^ ^5^D_0_–^7^F_1_ emission drops more significantly in the Eu^3+^-exchanged NCs (1:25) than in the nuclei when changing the excitation wavelength from 393 to 463.5 nm.

Temporal spectroscopic analysis provides critical information (Figure 4a,b). The relative intensity of the ^5^D_0_–^7^F_2_ to the ^5^D_0_–^7^F_1_ for the Eu^3+^-exchanged NCs (1:25) in the time-resolved emission spectra (TRES) decreases with the extended signal acquisition delay, both upon excitations of Eu^3+^ ^7^F_0_–^5^L_6_ (393 nm) and ^7^F_0_–^5^D_2_ transitions (463.5 nm). For the Eu^3+^-exchanged nuclei, the drop in relative intensity (represented by the R value: the ratio of the ^5^D_0_–^7^F_2_ and ^5^D_0_–^7^F_1_ luminescence intensity) with delay time is less steep (Figures S18 and 4c) compared to the NCs (1:25). In general, the spectral profile of the emission from a particular 4*f* excited level of a respective emitting Eu^3+^ species should have remained unchanged, as that of bulk Eu^3+^ residing at Wyckoff 4*a* position in the lattice of SYF (Figures S19 and 4c).^22^ These observations reveal more emitting Eu^3+^ species in the NCs (1:25) than in the nuclei. A close comparison of the spectral features of Eu^3+^ acquired from the TRES for the Eu^3+^-exchanged NCs with different signal acquisition delays (Figure S20) shows that the rise in relative intensity of the Eu^3+^ ^5^D_0_–^7^F_1_ over the ^5^D_0_–^7^F_2_ emission in the TRES primarily originates from the emergence of the bulk Eu^3+^ ^5^D_0_ luminescence. These spectroscopic findings enable us to decode the distinct structural evolution across the surface regions of NCs of different sizes (Figure 4d). For the large NCs with a cuboidal morphology, lattice distortion diminishes abruptly from the surface into the NC interior. At a short distance from the surface, bulk-like behavior of Eu^3+^ luminescence is already adopted. As a result, there is only a comparatively thin surface layer, where the cationic site deviates from the bulk with a sharp gradient in lattice relaxation (Figure 4d.1.). In this case, the exchanged Eu^3+^ in NCs (1:25) can transform into sites with less distortion by slight diffusion inward, with its luminescence being more regulated by the transition selection rule. In contrast, the gradient in lattice relaxation from the surface into the NC interior is less for the small spheroidal nuclei, creating a relatively thick surface layer (Figure 4d.2.). Accompanied by a low energy barrier for exchange, this thick surface layer greatly promotes Eu^3+^ exchange, leading to the bright luminescence observed in the Eu^3+^-exchanged SYF nuclei.

**Figure 4:**
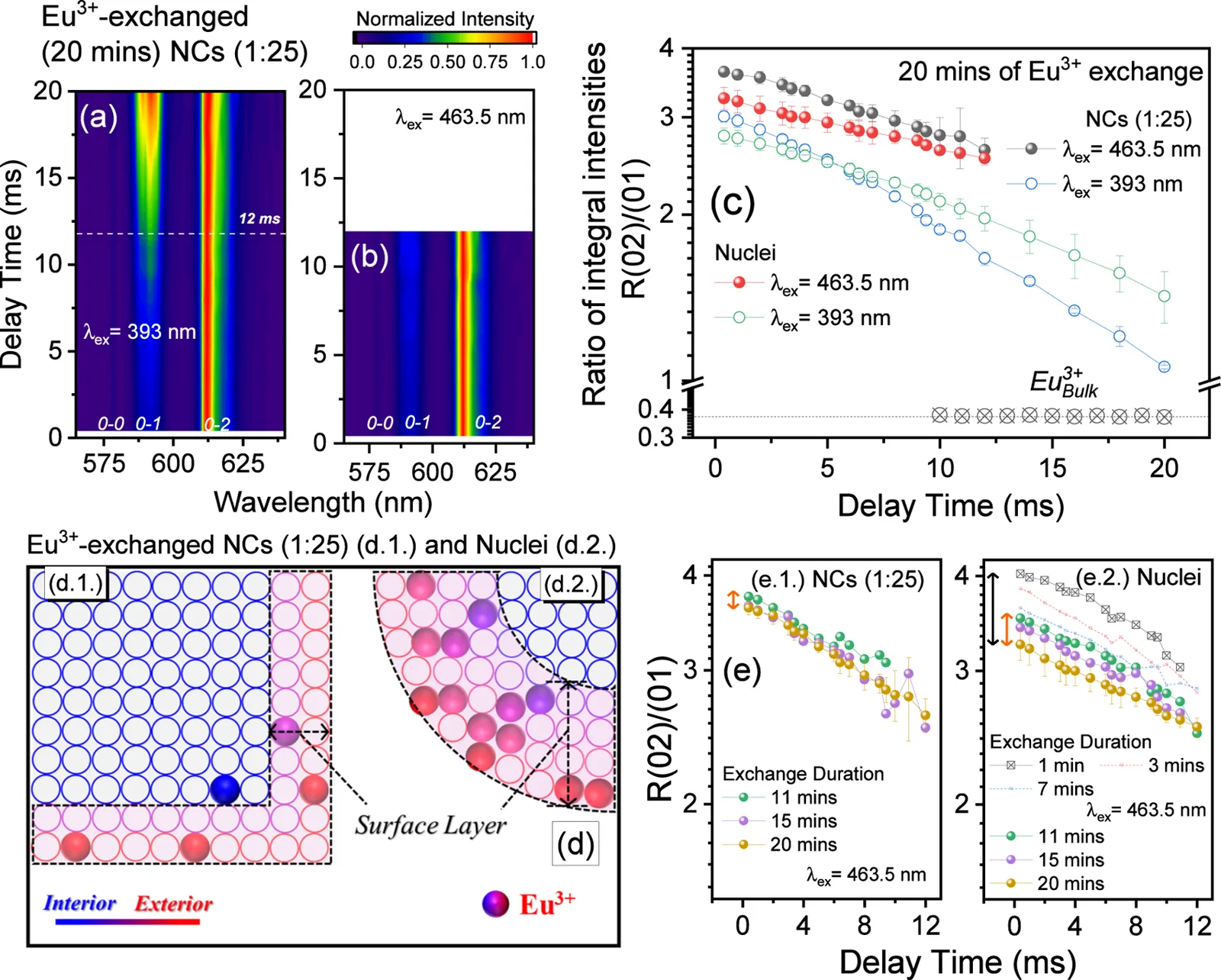
(**a**) Color contours of the normalized TRES of the Eu^3+^-exchanged (20 min) NCs (1:25) upon excitations of the Eu^3+^ ^7^F_0_–^5^L_6_ (393 nm) and (**b**) ^7^F_0_–^5^D_2_ transitions (463.5 nm), respectively. (**c**) Dependences of the integral intensity ratio R(02)/(01) of the Eu^3+^ ^5^D_0_–^7^F_2_ and ^5^D_0_–^7^F_1_ luminescence (the R value), on the signal acquisition delay in the TRES of the Eu^3+^-exchanged (20 min) nuclei and NCs (1:25) upon different excitation wavelengths. The corresponding *R* value of Eu^3+^ substituting at the Wyckoff 4*a* lattice site (termed bulk Eu^3+^) in the lattice of SYF is provided for comparison. Data deviations from multiple independent experiments under identical conditions are provided as error bars. (**d**) Chart schematizing the surface exchange of Eu^3+^ and different surface features of the Eu^3+^-exchanged NCs of varying sizes. (**e**) Dependences of the *R* value on the signal acquisition delay in the TRES of the Eu^3+^-exchanged nuclei and NCs (1:25) with different exchange durations upon 463.5 nm excitation. The extent of variation of the temporal-related trends against exchange durations in each case is indicated by arrows. Data points are connected by lines to guide the eye, but they do not represent a fit.

We further analyze the temporal spectroscopic features of Eu^3+^-exchanged NCs with varying exchange durations (Figures 4e, S21, and S22). Increasing the exchange duration has a relatively minor impact on the delay time-dependent spectral profiles of Eu^3+^ luminescence (represented by the *R* value) in the Eu^3+^-exchanged NCs (1:25) (Figure 4e.1). In contrast, the impact appears to be profound for the Eu^3+^-exchanged nuclei, which causes an overall decrease in this ratio (Figure 4e.2). Similar observations were made when changing the excitation wavelength from 463.5 to 393 nm (Figures S23–S25). These results support the finding that the extent of Eu^3+^ substitution at the thin surface layer of large-sized NCs (1:25) is severely limited, as evidenced by the negligible variance with exchange durations. This behavior contrasts with that of small nuclei, where the trends show greater variation with exchange durations.

## Conclusion

This work challenges the prevailing view by highlighting that surface evolution during NC growth plays a vital role in governing the luminescence of Ln^3+^-doped NCs, as unveiled by using surface-exchanged Eu^3+^ as a luminescent probe in the SYF NCs of different sizes and morphologies. Steady-state spectral analysis of the series of Eu^3+^-exchanged NCs with varying exchange durations shows, contrary to expectation, a stark drop in the luminescence intensity of NCs grown in size. Atomic-resolution electron microscopy imaging traces this behavior to surface evolution, characterized by a gradual shift in the exposed crystal facets from {111} to {200}, as well as a morphological transformation from spheroidal to cuboidal. Temporal spectroscopic analyses demonstrate that Eu^3+^ exchange at the thin surface layer of large-sized SYF NCs is severely limited, resulting in the stark drop in luminescence.

More fundamentally, this work demonstrates that the surface structure of NCs controls their photophysical properties. As NCs grow, the change in surface generates a more symmetric local environment around the Ln^3+^ ions that, in turn, affects how efficiently the Ln^3+^ ions emit light. Areas of greater lattice distortion not only reduce the energy barrier for exchange and allow for a high substitution extent of optically active centers in the system but also lift the restriction of selection rules for electronic transitions, thereby improving the luminescence of materials. These insights highlight the importance of crystal growth-induced surface structure evolution, particularly the role of lattice distortion-dependent surface reaction kinetics at the nanoscale, and open opportunities for developing versatile nanomaterials suitable for frontier applications. Future efforts will focus on developing strategies to control surface structure for specific applications and improve the luminescence efficiency of materials via structure optimization and engineering.

## Methods

### Materials Synthesis

#### Chemicals

SrCO_3_ (99.9%), trifluoroacetic acid (99%), oleic acid (OA, 90%), 1-octadecene (ODE, 90% tech grade), oleylamine (OM, tech grade), and europium acetate hydrate (99.99%) were purchased from Sigma-Aldrich, Merck, Steinheim, Germany. Yttrium oxide (Y_2_O_3_, 99.99%) was purchased from HEFA Rare Earth Canada Co. Ltd., Richmond, British Columbia, Canada. Sodium oleate (>97.0%) was purchased from TCI, Tokyo Chemical Industry, Tokyo, Japan. Cyclohexane, hexane, and absolute ethanol were purchased from VWR, Denmark. All chemicals were used as received without further purification.

#### Preparation of Strontium­(II) Trifluoroacetate (TFA-Sr) and Yttrium­(III) Trifluoroacetate (TFA-Y) Precursors

The TFA-Y (or TFA-Sr) precursor is prepared by following the procedure described in our previous work with mild modification.^22^ Briefly, 15 mmol Y_2_O_3_ powder, 10 mL 99% trifluoroacetic acid and 7.5 mL distilled water are added into a 250 mL 2-neck round-bottom flask. This mixture is heated to 90 °C under continuous stirring for 2 h. A condenser is used to avoid the possible evaporation loss of trifluoroacetic acid. An optically transparent solution is obtained. After that, the flask is put under dynamic vacuum at a Schlenk line to evaporate remaining water and trifluoroacetic acid, and a white powder of 30 mmol TFA-Y is obtained. Next, 45 mL OA and 45 mL ODE are added into the flask, yielding a concentration of TFA-Y to 0.33 mmol/mL. This mixture is then put under vacuum and slowly heated to 90 °C under continuous stirring and kept for 30 min. Subsequently, the temperature is increased to 120 °C and held for 30 min, enabling all TFA-Y to disperse into the oil phase, and an optically transparent dispersion is formed. After cooling to room temperature (RT), the TFA-Y precursor dispersion is transferred into glass vials for later use.

For TFA-Sr, 60 mmol SrCO_3_ powder, 10 mL 99% trifluoroacetic acid and 7.5 mL distilled water are added into a round-bottom flask. The reaction starts immediately after the addition of acid, and all SrCO_3_ powder completely dissolves within 10 min under sonication in an ultrasonic bath. The flask is then put under dynamic vacuum to evaporate the remaining water and trifluoroacetic acid, and a white powder of 60 mmol TFA-Sr is obtained. Next, 90 mL OA and 90 mL ODE are added into the flask, yielding a concentration of TFA-Sr to 0.33 mmol/mL. This mixture is then put under vacuum and slowly heated to 95 °C and held for 30 min, and an optically transparent dispersion is formed. After cooling to RT, the TFA-Sr precursor dispersion is transferred into glass vials for later use.

#### Preparation of Eu­(III) Oleate Used for Cation Exchange

5 mmol europium acetate hydrate and 15 mL OA and 15 mL ODE are added into a 100 mL flask. The flask is then put under vacuum and slowly heated to 150 °C and held for 30 min to react all europium acetate, and an optically transparent solution is formed with the concentration of Eu­(III) oleate to 0.17 mmol/mL. After cooling to RT, the Eu­(III) oleate dispersed in 1-octadecene is transferred into vials for later use.

#### Synthesis of Sr_2_YF_7_ (Abbreviated as SYF) Nuclei

The host SYF nuclei are prepared by the method of thermal decomposition described in our previous work with mild modifications.^22^ Typically, 4 mmol TFA-Sr (12 mL) and 2 mmol TFA-Y (6 mL) precursor dispersions are added into a 100 mL 3-neck round-bottom flask according to the nominal chemical formulas of Sr_2_YF_7_. An additional 3 mL OA, 17 mL ODE, and 4 mL OM are added to the flask to reach a total amount of 12 mL OA, 26 mL ODE, and 4 mL OM. Next, the mixture is put under vacuum at a Schlenk line and heated to 90 °C at roughly 300 rpm stirring. The mixture is kept at 90 °C for 10 min, and then Ar and vacuum are cycled three times to remove any residual water and oxygen. The solution is subsequently heated to 300 °C under continuous gentle Ar flow in roughly 15 min. The reaction is kept at 300 °C for 30 min. Then the heating mantle is removed, and the mixture is cooled to RT naturally. The viscous solution obtained is transferred to four 50 mL centrifuge tubes, and roughly 40 mL freshly mixed solvent (hexane/absolute ethanol (v/v) = 1:2) is added into each tube to precipitate the product NCs. After centrifugation at 7000 rpm for 10 min (VWR Mega Star 600R, with HIGHConic III Rotor), a sediment is obtained at the bottom of tube. The washing process involves dispersing these products into 10 mL cyclohexane, adding 25 mL absolute ethanol to precipitate, and centrifuging at 7000 rpm. After the washing step, nominally 2 mmol of neat SYF nuclei are obtained. These product nuclei are dispersed and stored in 20 mL cyclohexane for later use.

#### Synthesis of SYF NCs of Varying Sizes (NCs (1:3), NCs (1:5), and NCs (1:25))

The preparation of host SYF NCs of varying sizes is enabled by layer-by-layer growth through hot-injecting the trifluoroacetate precursors with identical chemical composition as used for the nuclei preparation, with the SYF nuclei serving as crystal seeds. Different amounts of TFA-Sr and TFA-Y precursor dispersions are initially mixed with each other (v/v = 2:1) under continuous stirring at RT to reach the concentration of SYF precursor to 0.11 mmol/mL for later use. In a typical experiment of NCs (1:3), a 1 mL cyclohexane dispersion nominally containing 0.1 mmol SYF nuclei is added into a 100 mL 3-neck round-bottom flask with 5 mL OA and 5 mL ODE. This mixture is put under vacuum and heated to 90 °C at roughly 300 rpm stirring for 5 min. After that, Ar and vacuum are cycled three times to remove the remaining cyclohexane and oxygen, and the solution is heated to 300 °C under continuous gentle Ar flow in 15 min. To trigger crystal growth, 2.7 mL well-mixed precursor is transferred into an injection syringe and injected through a septum into the flask when the temperature of reaction system reaches 300 °C. The injection amount is set to be 1 mL for each injection, and the interval time between each injection is a 3 min period. When the remaining volume of precursor is less than 1 mL, inject it all at once in the next injection. Depending on the different amounts of precursors used, different reaction times are required. After the completion of the precursor injection (9 min in this case), the solution continues to react for 5 min further and then cooled to RT naturally. The same sample precipitation and washing procedure as for the nuclei (see above) is adopted, and the product NCs (1:3) are dispersed and stored in cyclohexane.

For NCs (1:5) and NCs (1:25), 4.5 and 22.7 mL well-mixed precursors are used for crystal growth, respectively, with other experimental conditions constant.

#### Experimental Details for the High-temperature Cation Exchange of Eu^3+^ at the Surface of NCs of Varying Sizes with Different Exchange Durations and Successive Specimen Sampling

In a typical experiment, 2 mmol SYF NCs are prepared following the procedure mentioned above and dispersed into 10 mL cyclohexane. Next, a 5 mL cyclohexane dispersion nominally containing 1 mmol NCs is added into a 100 mL 3-neck round-bottom flask with 10 mL OA and 10 mL ODE. This mixture is put under vacuum to remove the cyclohexane carefully and heated to 90 °C at roughly 300 rpm stirring for 5 min. After that, Ar and vacuum are cycled three times to remove the moisture and oxygen, and the solution is heated to 300 °C under continuous gentle Ar flow in 15 min. The reaction medium is kept at 300 °C for 5 min to reach a thermally stable state. Then, 0.2 mmol Eu­(III) oleate dispersed in 1-octadecene (1.2 mL) is hot-injected through a septum into the flask in one shot, which triggers the Eu^3+^ exchange at the surface of NCs. The moment of injection is defined as a time reference of 0 min. Aliquots (3 mL each time) are then extracted from the reaction mixture with the exchange durations of 1, 3, 7, 11, 15, and 20 min and stored in the vials. The rest of the product in the flask is discarded, and the heating mantle is removed to let the flask cool to RT naturally. Next, these aliquots are transferred to 50 mL centrifuge tubes, and 30 mL freshly mixed solvent (hexane/absolute ethanol (v/v) = 1:2) is added into these tubes to precipitate the product NCs. After centrifugation at 7000 rpm for 10 min, the NCs settle at the bottom of tubes. The supernatant liquid in each tube is collected for later spectral measurement. The sample washing process involves dispersing these products into 5 mL cyclohexane, adding 15 mL absolute ethanol to precipitate, and centrifuging at 7000 rpm. After the washing step, nominally 0.15 mmol of Eu^3+^-exchanged NCs of different exchange durations are obtained each. These product NCs are dispersed and stored in 2.5 mL cyclohexane for later measurement.

For the additional experiments of the temperature-dependent Eu^3+^ exchange at the surface of NCs of varying sizes, only the exchange temperature was changed during the experiments, with other experimental conditions constant.

### Characterization

The phase purity of samples was examined on a STOE STADI P Powder diffractometer operated at 40 kV and 40 mA, equipped with Ag Kα1 monochromatic radiation in transmission Debye–Scherrer geometry with a DECTRIS MYTHEN 1 K detector using a rotating 0.5 mm Lindemann glass capillary sample holder. Infrared spectra of samples were measured using a Bruker Alpha-P ATR spectrometer (Bruker AXS, Karlsruhe, Germany). Thermogravimetric analyses of samples were performed with a TG 449 F3 Jupiter (Netzsch, Selb, Germany). Measurements were carried out in aluminum oxide crucibles at a heating rate of 10 °C/min, using argon as the purge gas at a flow rate of 40 mL/min. Transmission electron microscopy (TEM) and high-angle annular dark-field scanning transmission electron microscopy (HAADF-STEM) images of NCs were taken on a transmission electron microscope (TALOS F200X, FEI Company, USA) equipped with a TWIN lens system, an X-FEG electron source and a CETA 16 M camera. The accelerating voltage was set to 200 kV. HAADF–STEM analyses were performed using a nanoprobe with a spot size of #7, a convergence angle of 10.5 mrad, and collection semiangles of 38−200 mrad. The atomic-resolution STEM–HAADF imaging of single NCs was assisted by the method of drift-corrected frame integration (DCFI). TEM images of samples were processed in ImageJ. Elemental analyses were performed by energy-dispersive X-ray spectroscopy (EDS) mapping recorded in STEM mode. Emission and excitation spectra of samples dispersed in cyclohexane were measured at RT on a combined fluorescence steady-state and lifetime spectrometer (Edinburgh Instruments, FLS1000), equipped with a 450W xenon lamp and a 60 W μF2 flash lamp with a pulse width of 1–2 μs as the excitation sources, respectively, and a thermoelectrically cooled (−22 °C) photomultiplier for detection. The collected spectral signals of the samples have undergone corrections, as has the background signal subtraction. The photoluminescence quantum yield of the sample was measured on the same FLS1000 spectrometer by using a PTFE-coated integrating sphere. The average lifetime of Eu^3+^ ^5^D_0_ luminescence is estimated by the formula: $\tau_{average}=\int_{0}^{\infty }\frac{I\left(t\right)}{I\left(0\right)}dt$ , where *I­(*0*)* and *I­(t)* represent the initial intensity and that at time *t*, respectively.^30^

## Supplementary Material



## Notes

Supplemental spectral features of Eu^3+^-exchanged SYF nuclei and NCs (1:25) with different exchange durations, as well as those of the supernatant liquids collected from the precipitation of NCs, the full-sized TEM images and EDS elemental mapping of NCs of varying sizes, excitation/emission wavelength-selective steady-state and TRES spectra of Eu^3+^-exchanged NCs, and the numerical analyses of the variations of the Eu^3+^ emission profile in the TRES of the Eu^3+^-exchanged NCs (PDF)
