# Supplementary material for: Size-Dependent Surface Evolution Controls Luminescence in Ln3+-Doped Nanocrystals
Source: ACS Nano. 2026 Oct 23;20(44):29700–8. doi: 10.1021/acsnano.6c11081 (PMC13757963; doi:10.1021/acsnano.6c11081)
Supplement: Supplementary file 1 [file nn6c11081_si_001.pdf]

Supporting Information

Size-Dependent Surface Evolution Controls Luminescence in Ln^{3+}-Doped Nanocrystals

Rui Shi[1], Guillaume Bousrez[1], Ramkumar Venkatachalam[1], and Anja-Verena Mudring[1,2*]

[1] intelligent Advanced Materials, Department of Biological and Chemical Engineering and iNANO, Aarhus University, Aarhus C, 8000, Denmark

[2] Department of Physics, Umeå University, 901 87 Umeå, Sweden

*Corresponding Author: anja-verena.mudring@bce.au.dk (A.V.M.)

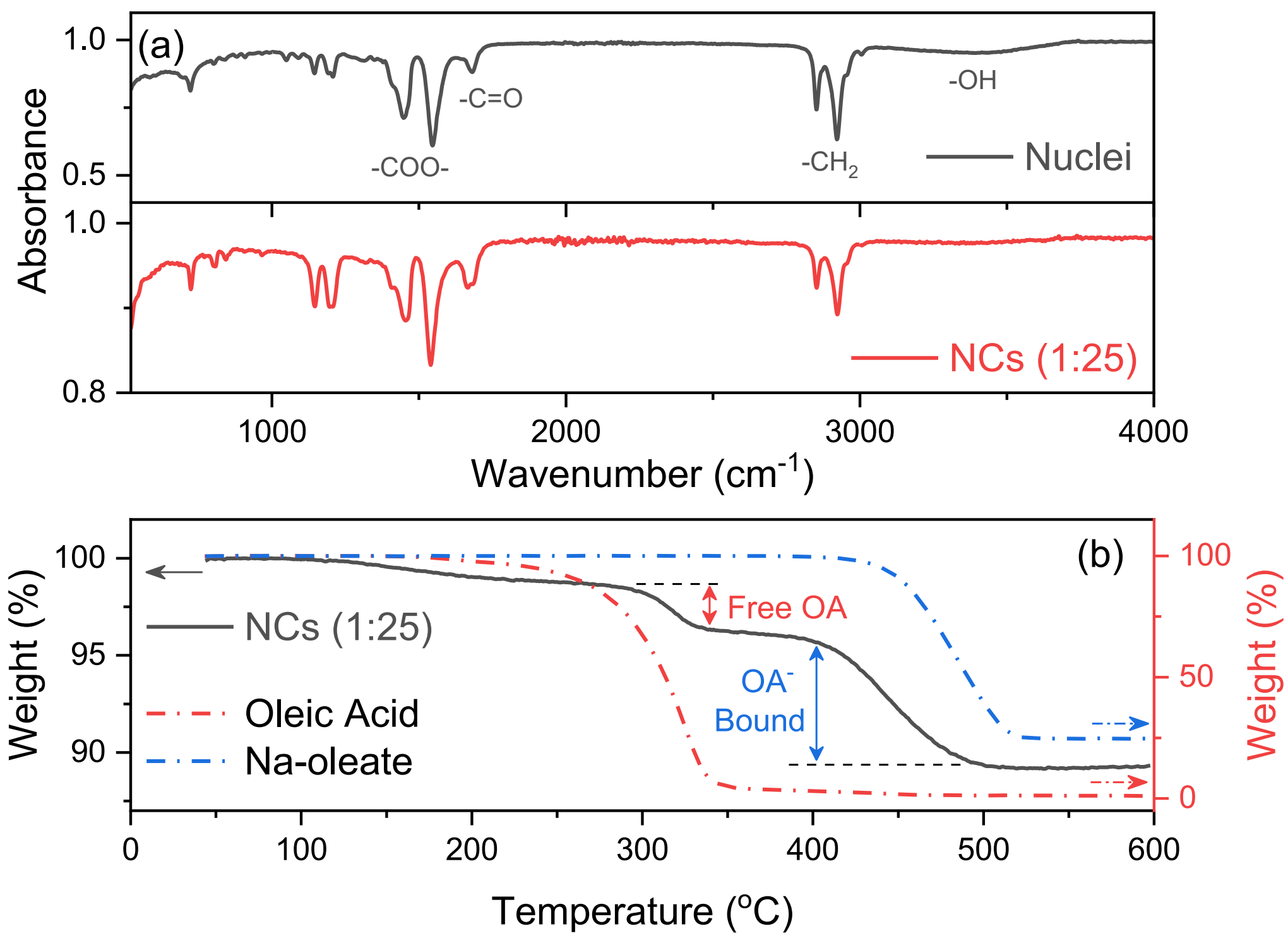

Figure S1. (a) Representative infrared spectra of SYF nuclei and NCs (1:25). The appearance of high-frequency vibrational modes of organic groups in the spectra of NCs of varying sizes shows surface binding of the long-chain oleic surfactant used during material synthesis. (b) Thermogravimetric analysis (TGA) trace recorded for the representative NCs (1:25) in comparison to neat oleic acid (OA) and sodium oleate. The TGA of NCs (1:25) shows a three-step weight loss. The first step corresponds to the removal of moisture, and the latter two are attributed to the thermal decomposition of free OA molecules and bound OA^-, the surfactants on the particle surface, respectively, by comparing the TG traces of neat OA and commercially available NaOA. This observation is consistent with the two-step decomposition mechanism of the surfactant, as previously observed for OA-capped nanocrystalline materials,[1] where it was suggested that the two steps were due to two layers of adsorbed surfactant.

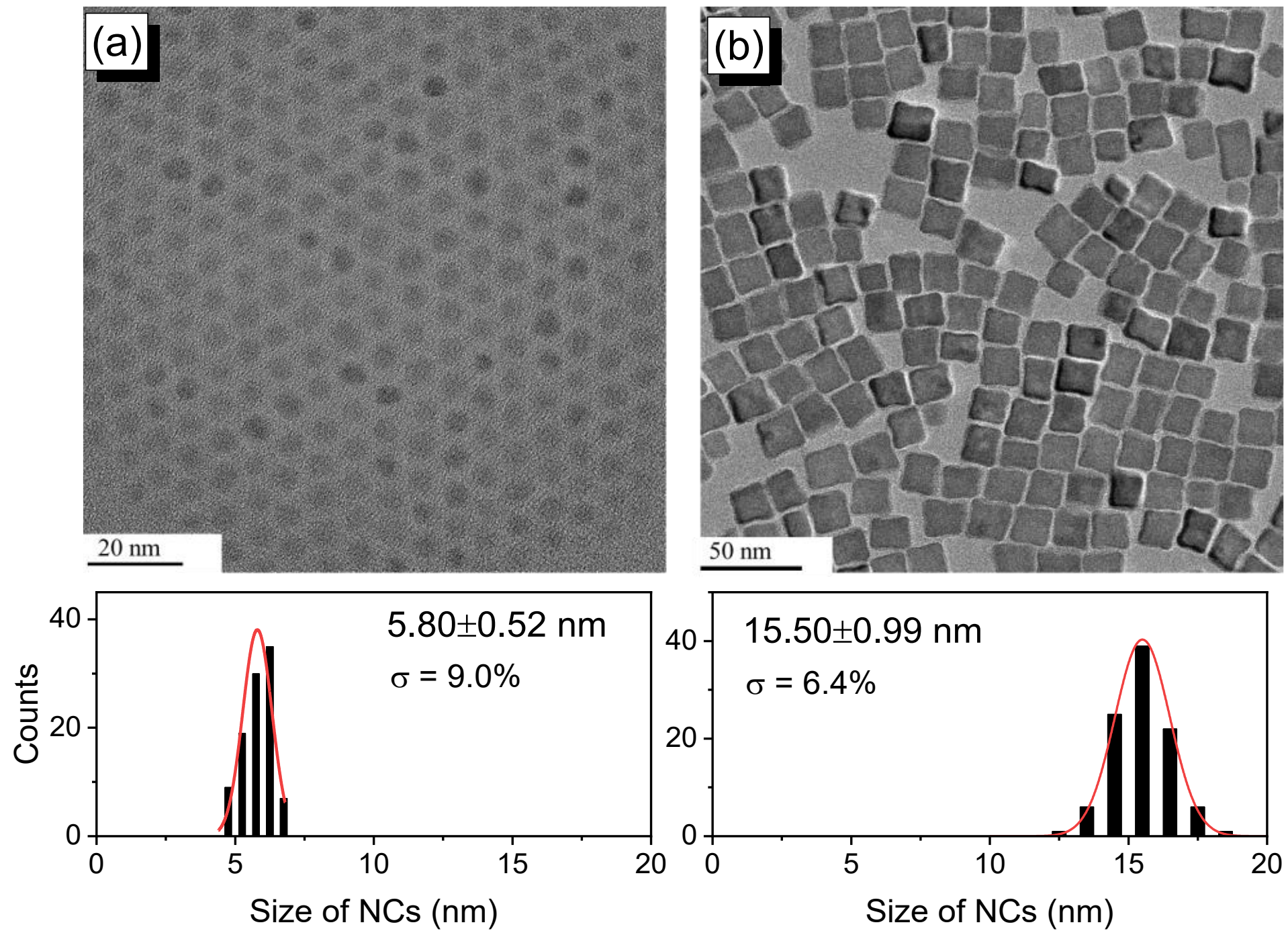

Figure S2. TEM images of the representative Eu^{3+}-exchanged (a) SYF nuclei and (b) NCs (1:25) and the corresponding size distributions.

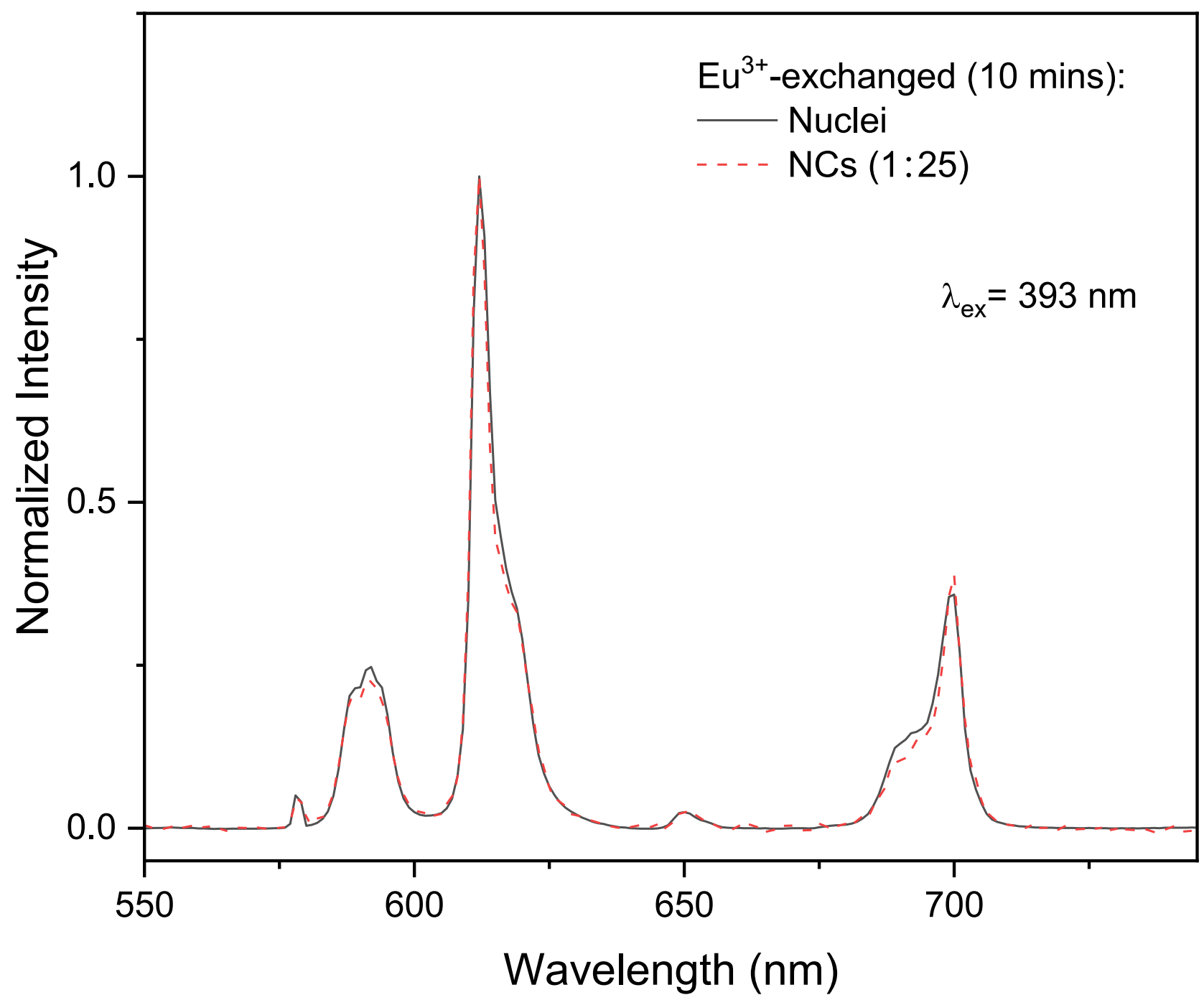

Figure S3. Comparison of the normalized emission spectra of the Eu^{3+}-exchanged (10 mins) SYF nuclei and NCs (1:25) upon 393 nm excitation.

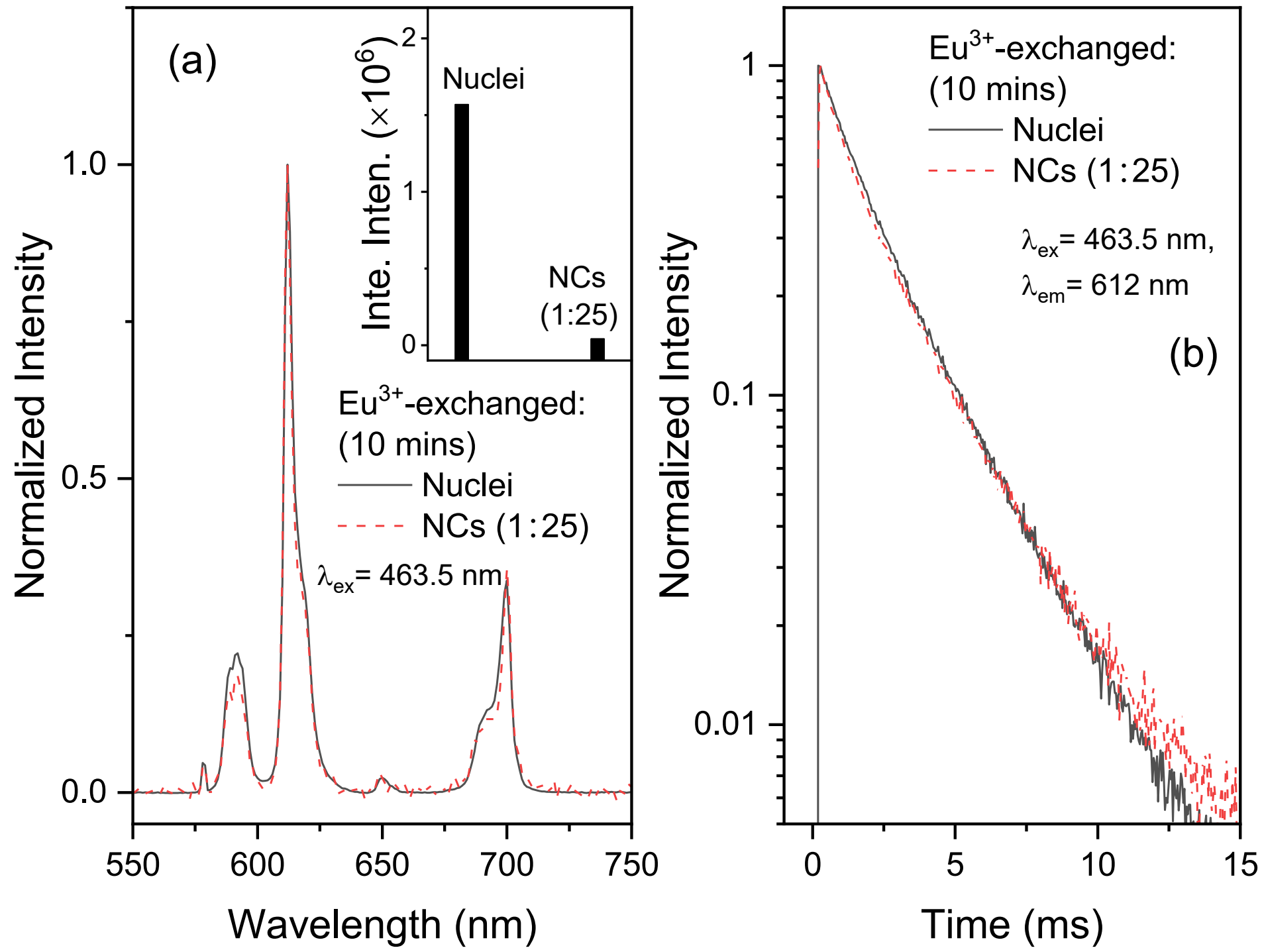

Figure S4. (a) Comparison of the normalized emission spectra of the Eu^{3+}-exchanged (10 mins) SYF nuclei and NCs (1:25) upon 463.5 nm excitation. The inset gives a comparison of the overall luminescence intensities of the samples. (b) Decay dynamics of the $^5D_0$–$^7F_2$ luminescence of Eu^{3+} in the Eu^{3+}-exchanged SYF nuclei and NCs (1:25) upon 463.5 nm excitation.

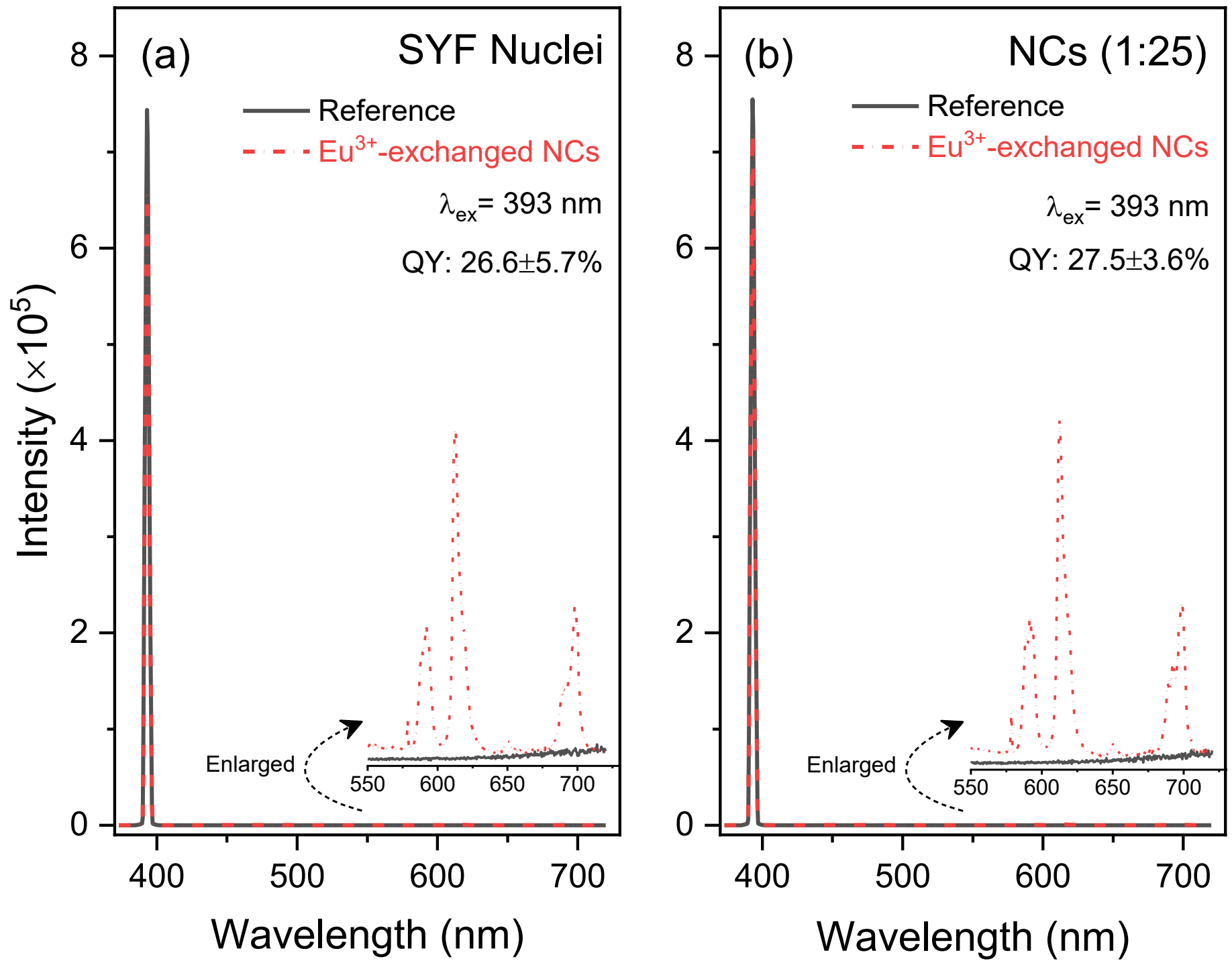

Figure S5. Photoluminescence quantum yield measurements of Eu^{3+}-exchanged (a) SYF nuclei and (b) NCs (1:25) upon 393 nm excitation.

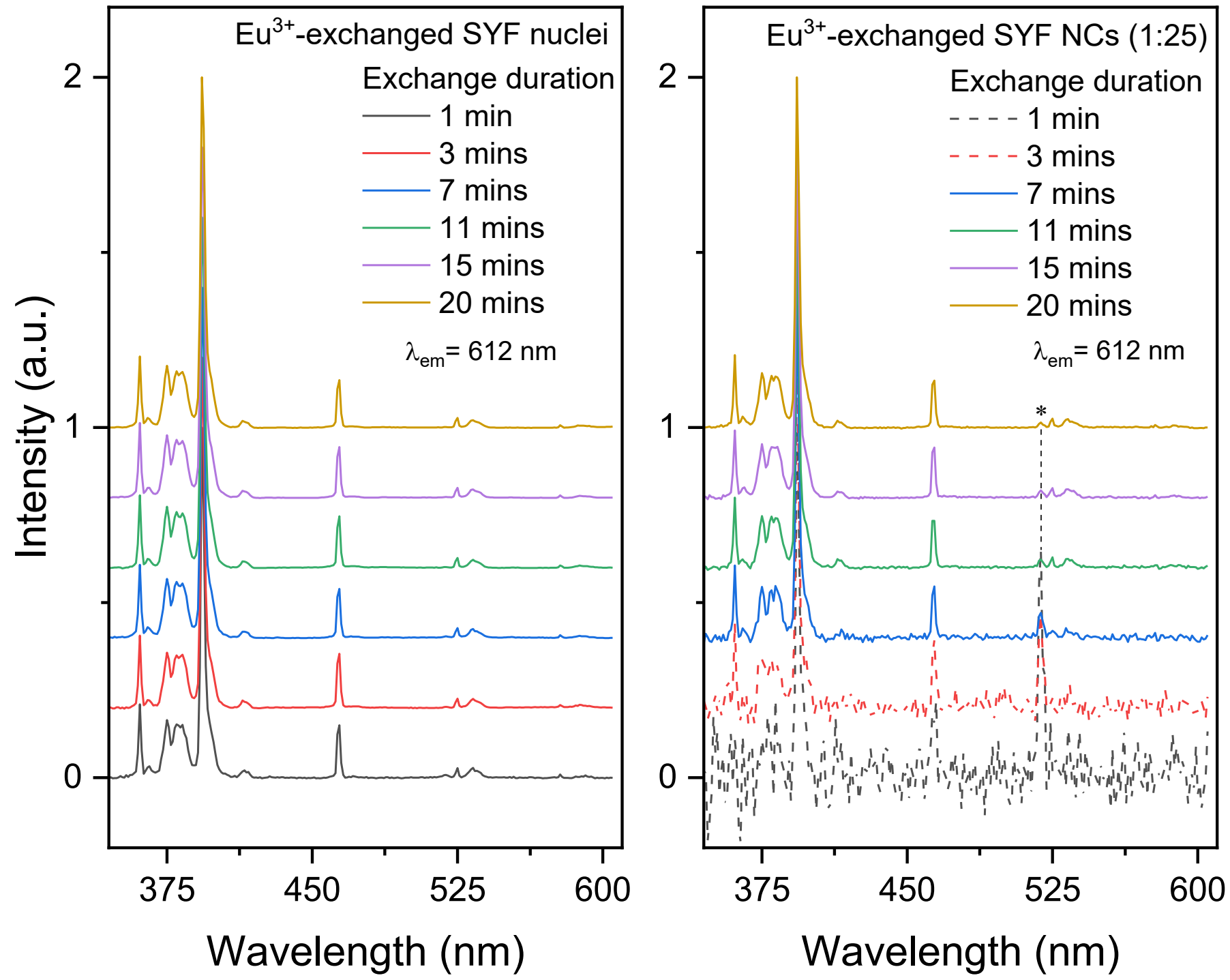

Figure S6. Normalized excitation spectra of the Eu^{3+}-exchanged SYF nuclei and NCs (1:25) with different exchange durations by monitoring 612 nm emission. The asterisk marks the signals originating from the light scattering of cyclohexane molecules.

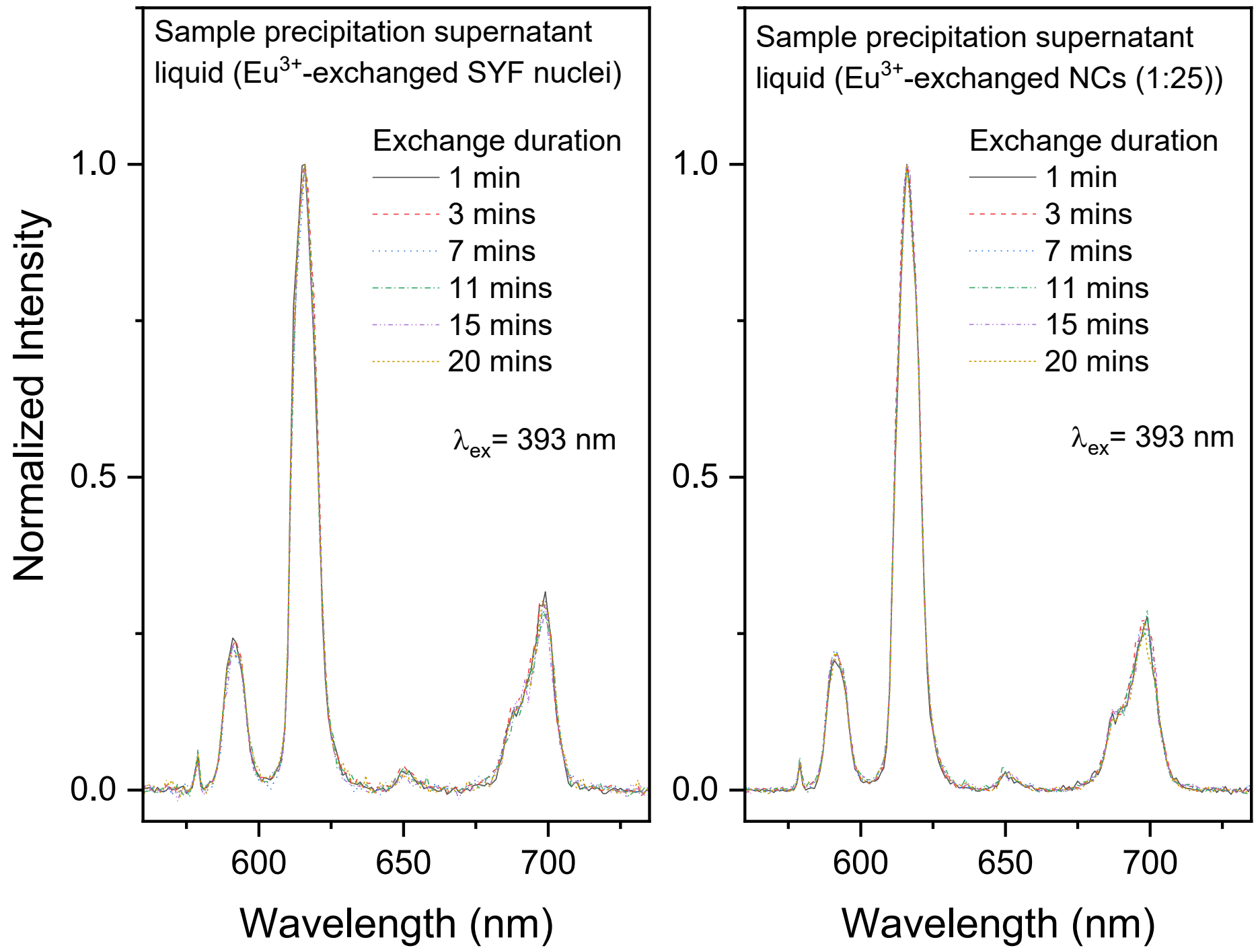

Figure S7. Normalized emission spectra of the supernatant liquids collected from the precipitation process of Eu^{3+}-exchanged SYF nuclei and NCs (1:25) with different exchange durations upon 393 nm excitation.

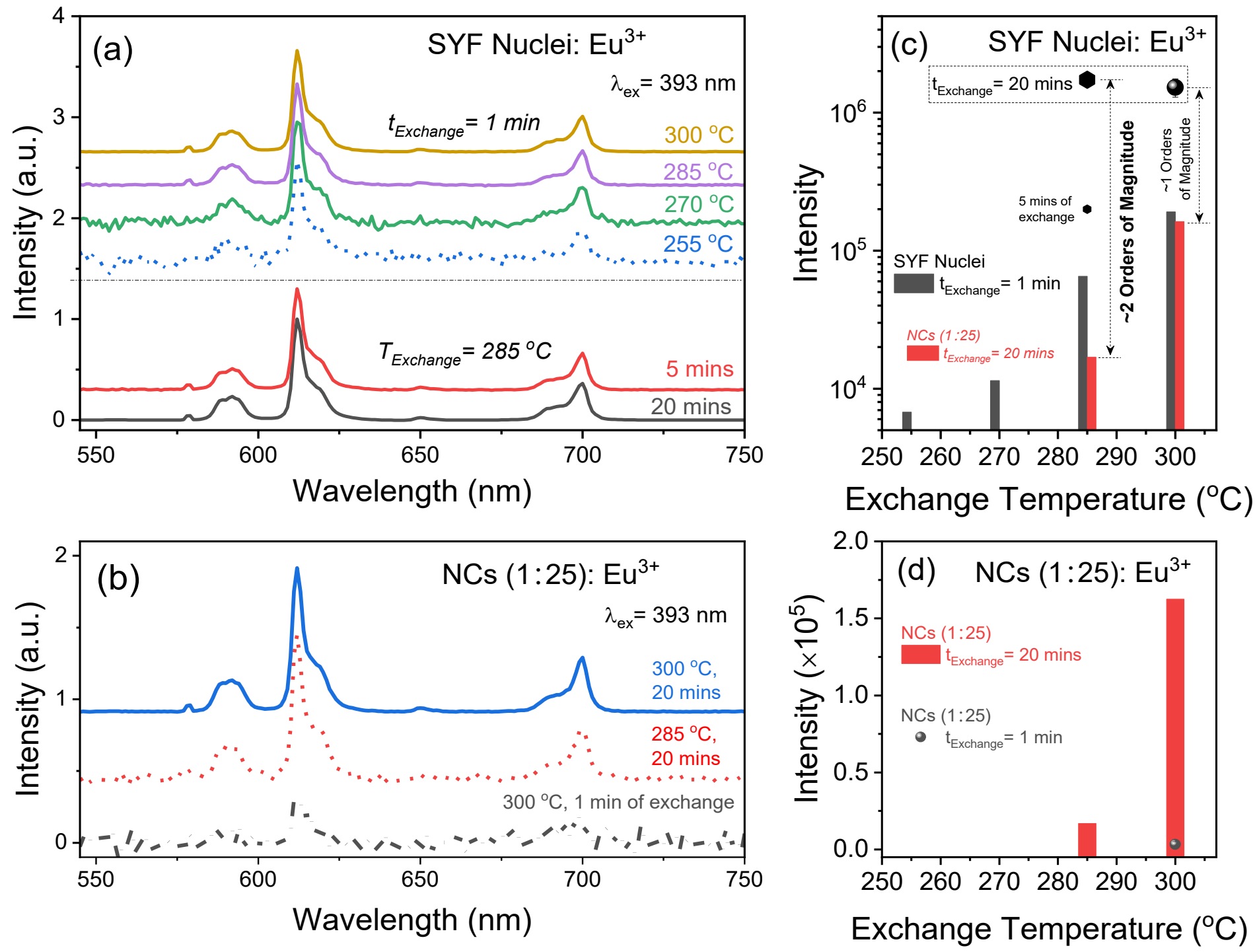

Figure S8. (a) Normalized emission spectra of Eu^{3+}-exchanged SYF nuclei and (b) NCs (1:25) prepared with different experimental conditions (*i.e.*, exchange temperature, $T_{Exchange}$, and duration, $t_{Exchange}$) upon 393 nm excitation, and (c, d) the corresponding experimental condition-dependent luminescence intensity of different samples. With a constant 1 min of $t_{Exchange}$, the luminescence intensity of the Eu^{3+}-exchanged nuclei increases quasi-exponentially with increasing $T_{Exchange}$ from 255 ºC to 300 ºC. After 20 mins of $t_{Exchange}$, the luminescence intensity of the Eu^{3+}-exchange nuclei at a low $T_{Exchange}$ of 285 ºC is close to that of the sample with Eu^{3+} exchanged at 300 ºC. Overall, Eu^{3+} exchange at the surface of SYF nuclei can be initiated at mild conditions ($T_{Exchange}$ low even to 255 ºC, with 1 min of $t_{Exchange}$), while the activation of Eu^{3+} exchange in the case of NCs (1:25) requires rather harsh conditions of high $T_{Exchange}$ and long $t_{Exchange}$.

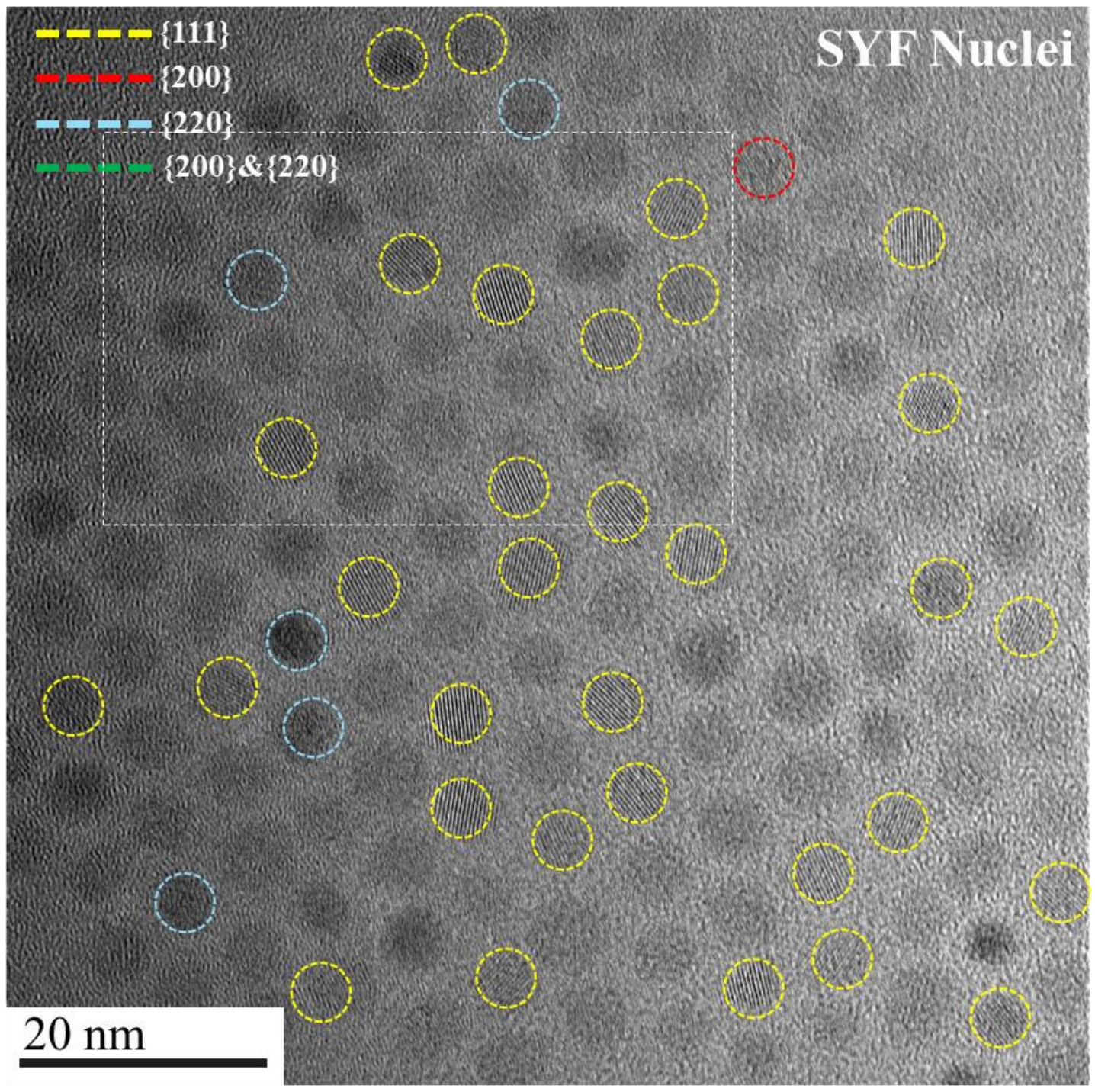

Figure S9. TEM image of SYF nuclei. Particles with clear lattice fringes observed in the TEM image are outlined in different colors depending on the sets of crystal planes associated with the lattice fringes: {111} in yellow, {200} in red, {220} in blue, and {200}&{220} in green. A white dashed box marks the cropped range of the image presented in the main article.

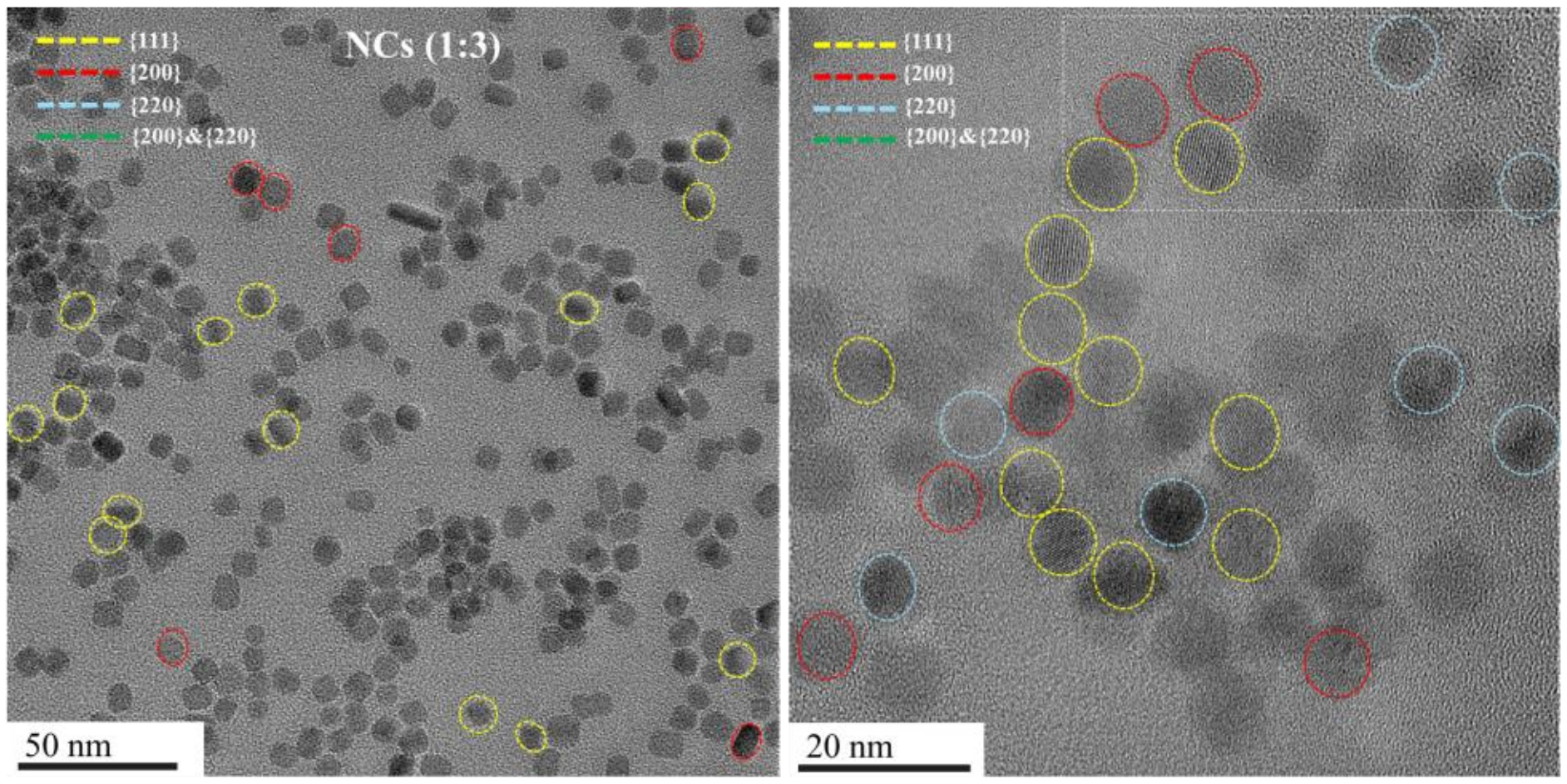

Figure S10. TEM images of SYF NCs (1:3). Particles with clear lattice fringes observed in the TEM images are outlined in different colors depending on the sets of crystal planes associated with the lattice fringes: {111} in yellow, {200} in red, {220} in blue, and {200} &{220} in green. A white dashed box marks the cropped range of the image presented in the main article.

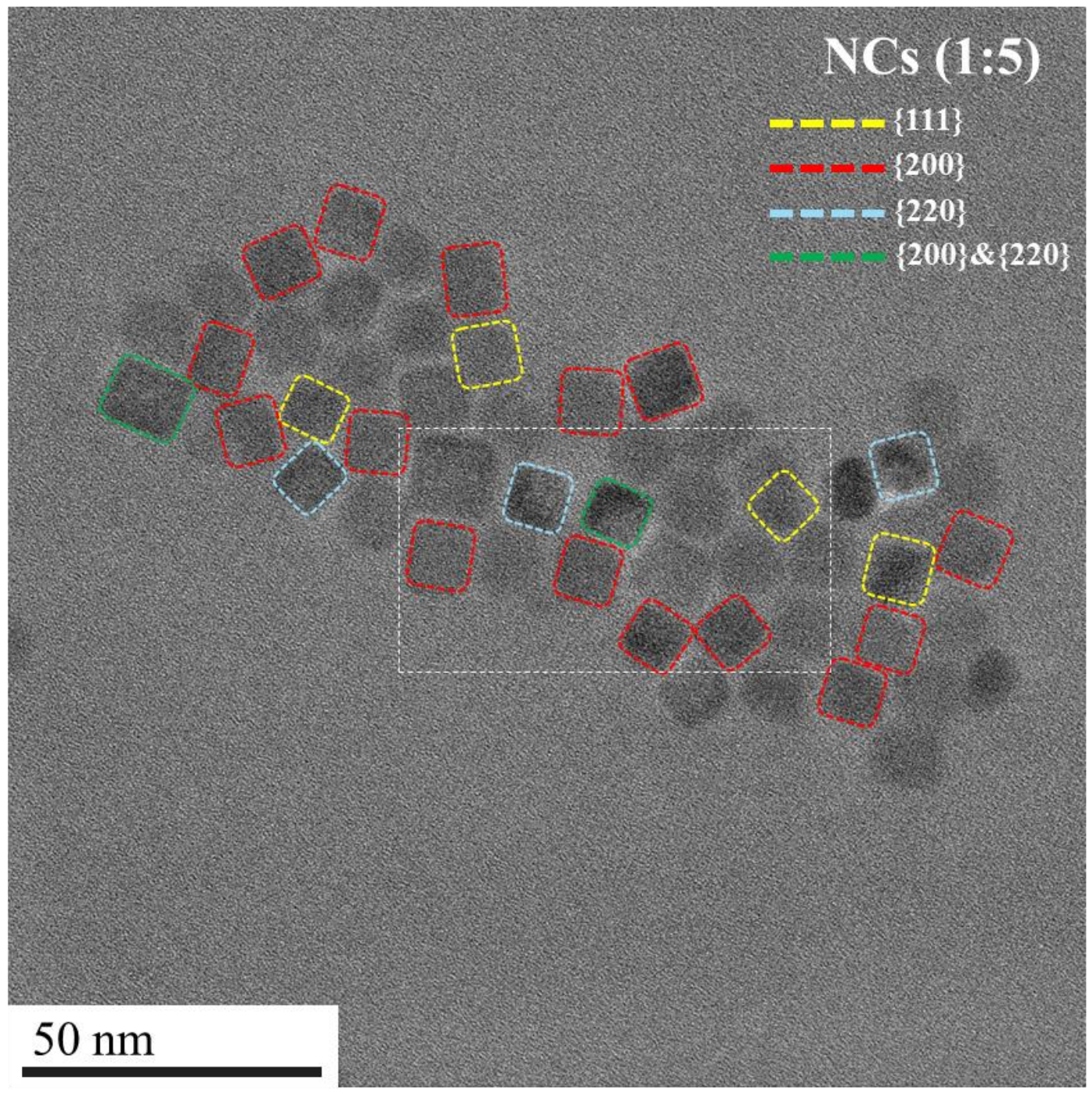

Figure S11. TEM image of SYF NCs (1:5). Particles with clear lattice fringes observed in the TEM image are outlined in different colors depending on the sets of crystal planes associated with the lattice fringes: {111} in yellow, {200} in red, {220} in blue, and {200} &{220} in green. A white dashed box marks the cropped range of the image presented in the main article.

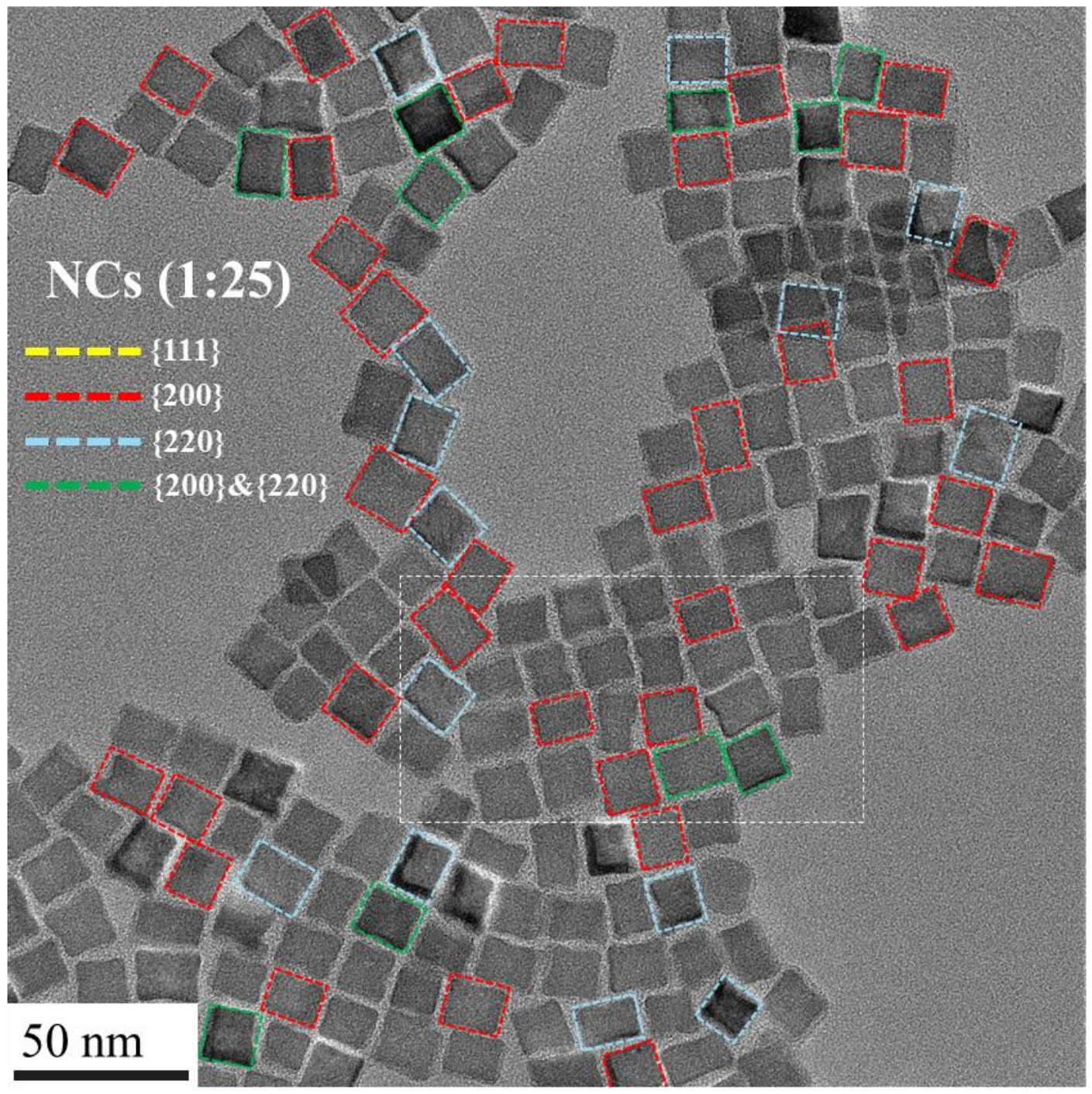

Figure S12. TEM image of SYF NCs (1:25). Particles with clear lattice fringes observed in the TEM image are outlined in different colors depending on the sets of crystal planes associated with the lattice fringes: {111} in yellow, {200} in red, {220} in blue, and {200} &{220} in green. A white dashed box marks the cropped range of the image presented in the main article.

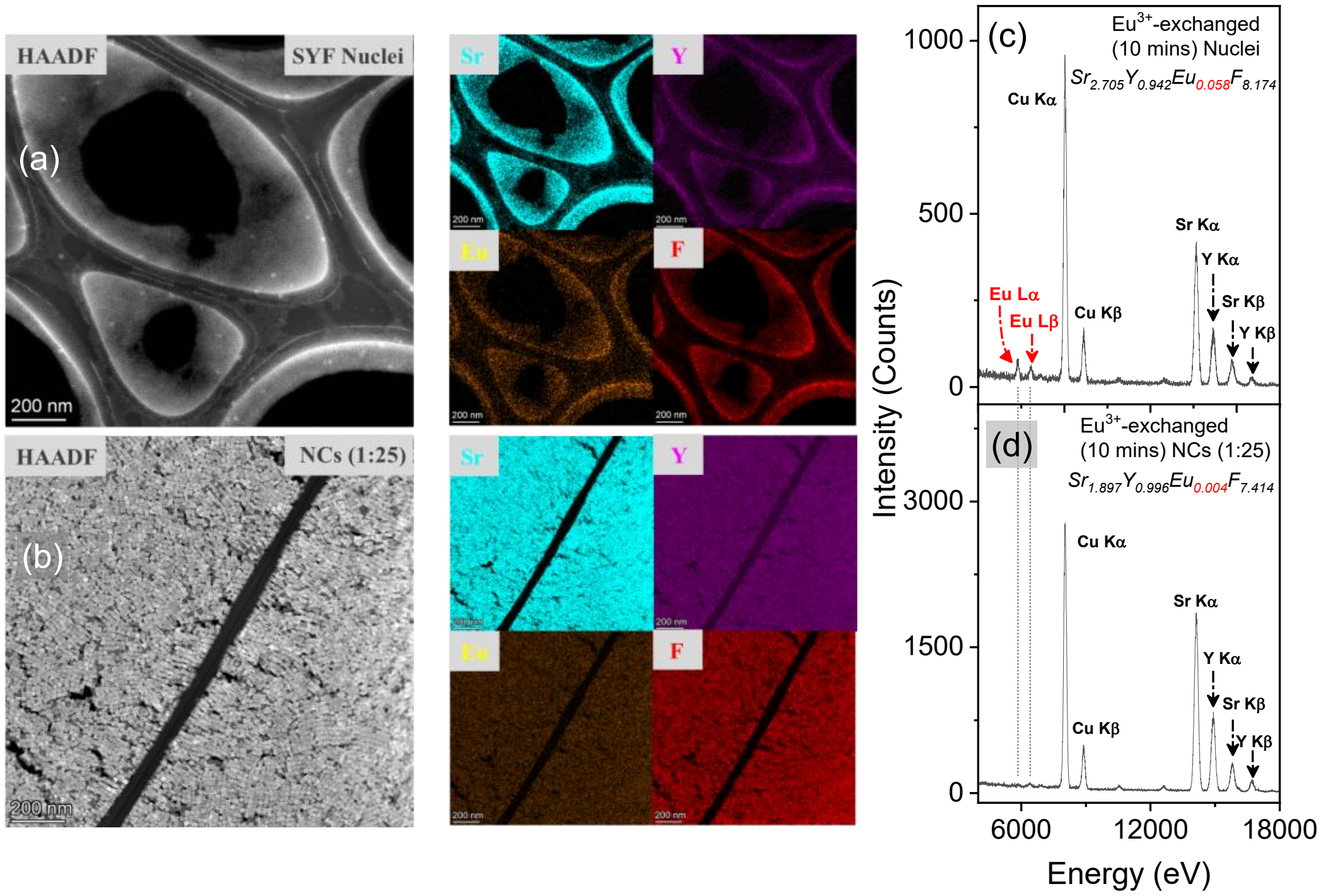

Figure S13. (a) STEM-HAADF image and EDS elemental mapping of Eu^{3+}-exchanged (10 mins of exchange) SYF nuclei and (b) NCs (1:25) ensembles. (c, d) Truncated EDS profile of Eu^{3+}-exchanged (10 mins of exchange) SYF nuclei and NCs (1:25) in the energy range of 4000-18000 eV, respectively. The peaks attributed to the Eu L_{α} and L_{β} lines are clearly observed in the spectrum of Eu^{3+}-exchange nuclei, in contrast to the almost absence of them in the case of Eu^{3+}-exchanged NCs (1:25). The EDS analysis of the estimated chemical compositions of these samples shows around one order of magnitude difference between the extent of Eu^{3+} substitution at the surface of NCs.

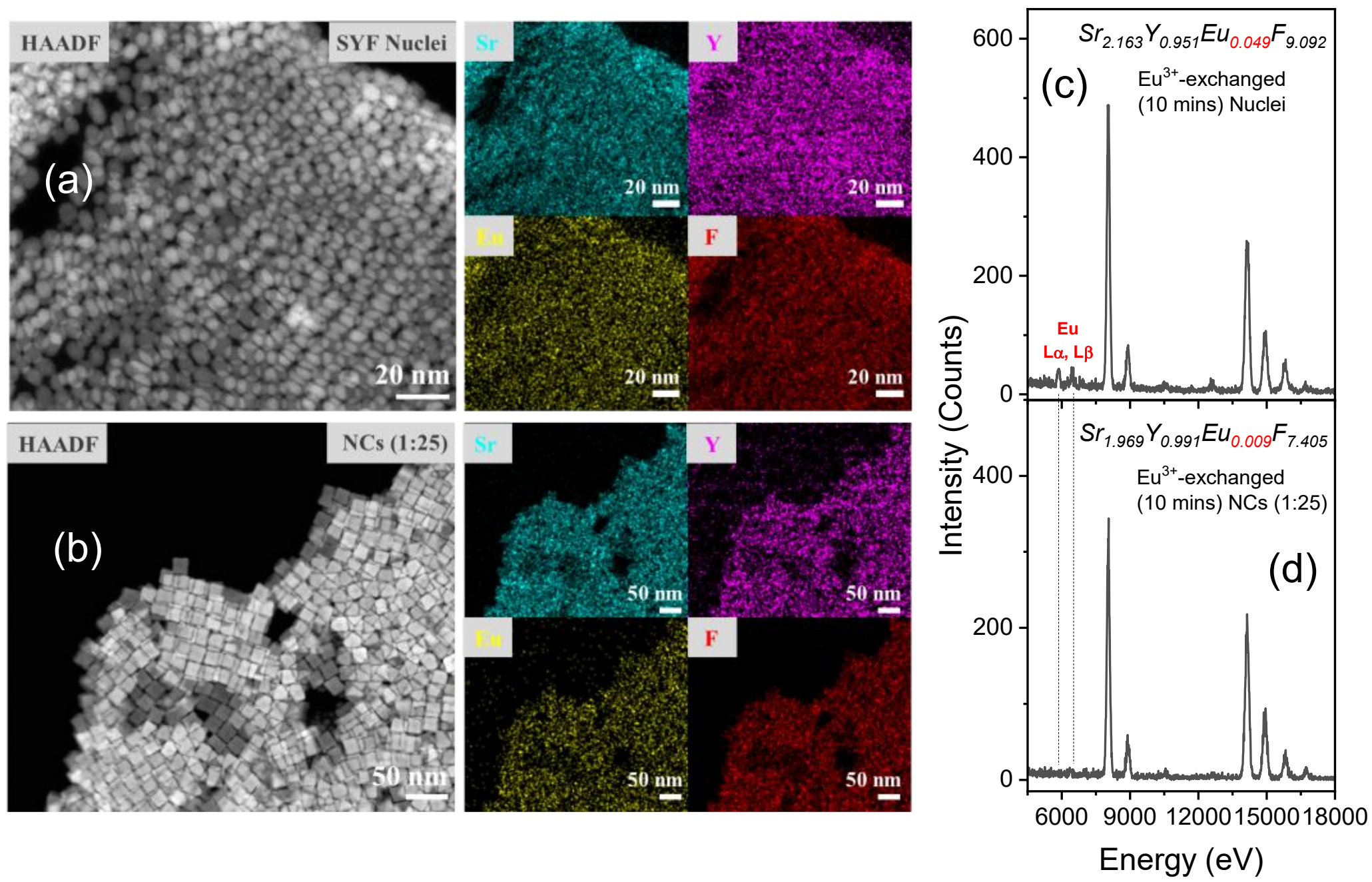

Figure S14. (a) STEM-HAADF image and EDS elemental mapping of Eu^{3+}-exchanged SYF nuclei and (b) NCs (1:25) ensembles at high magnification. (c, d) Truncated EDS profile of Eu^{3+}-exchanged SYF nuclei and NCs (1:25) in the energy range of 4000-18000 eV, respectively.

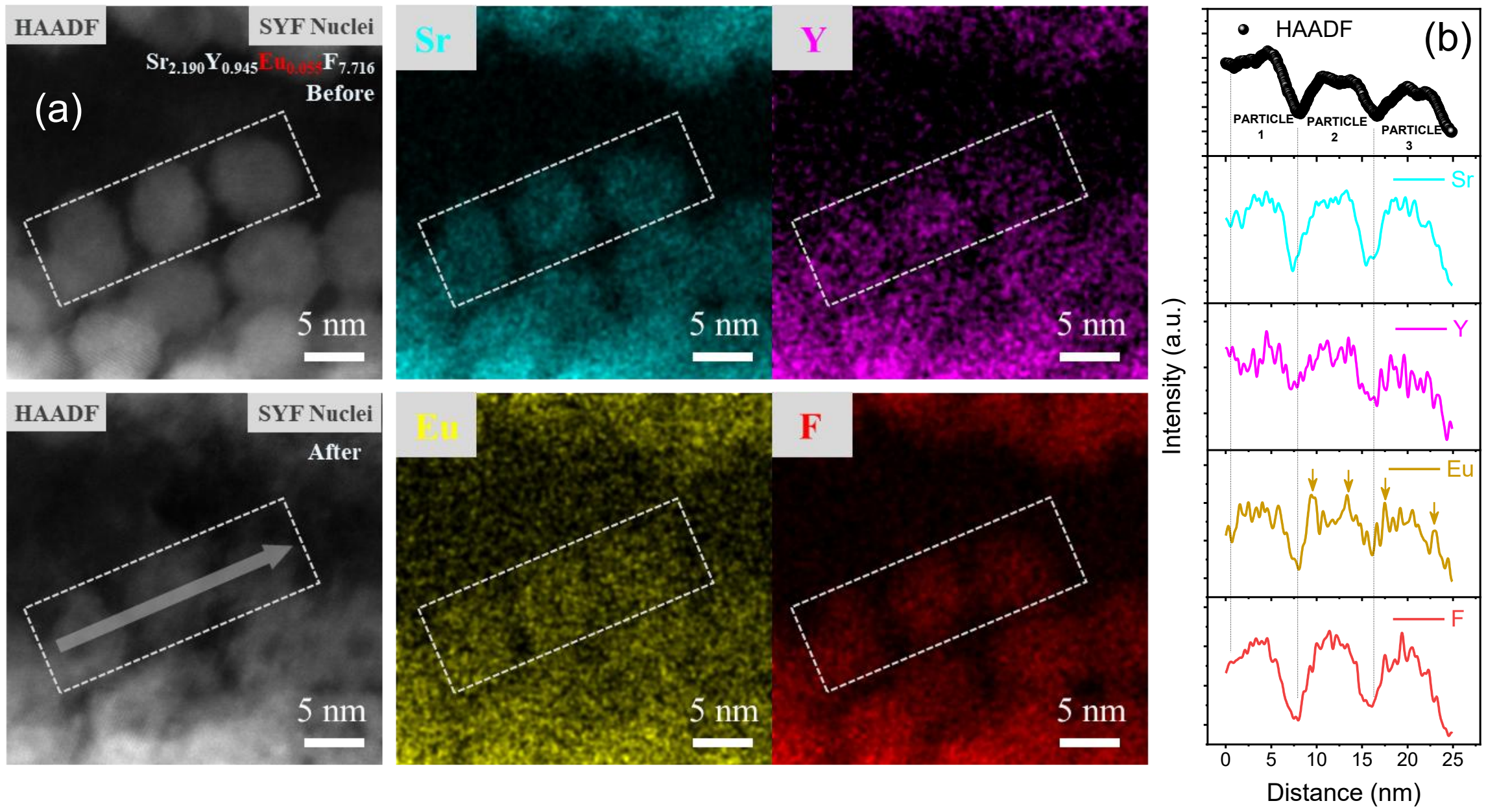

Figure S15. (a) Single particle-level STEM-HAADF image (before and after the measurement) and EDS elemental mapping of Eu^{3+}-exchanged SYF nuclei. The chemical composition of the sample as presented is estimated based on the EDS analysis. (b) HAADF and Sr, Y, Eu, and F elemental profiles of NCs at a single particle level, with the region of signal collection marked as the square in the figure. Some traces of the signal maxima of Eu element at the edge region of a single NC are marked by arrows.

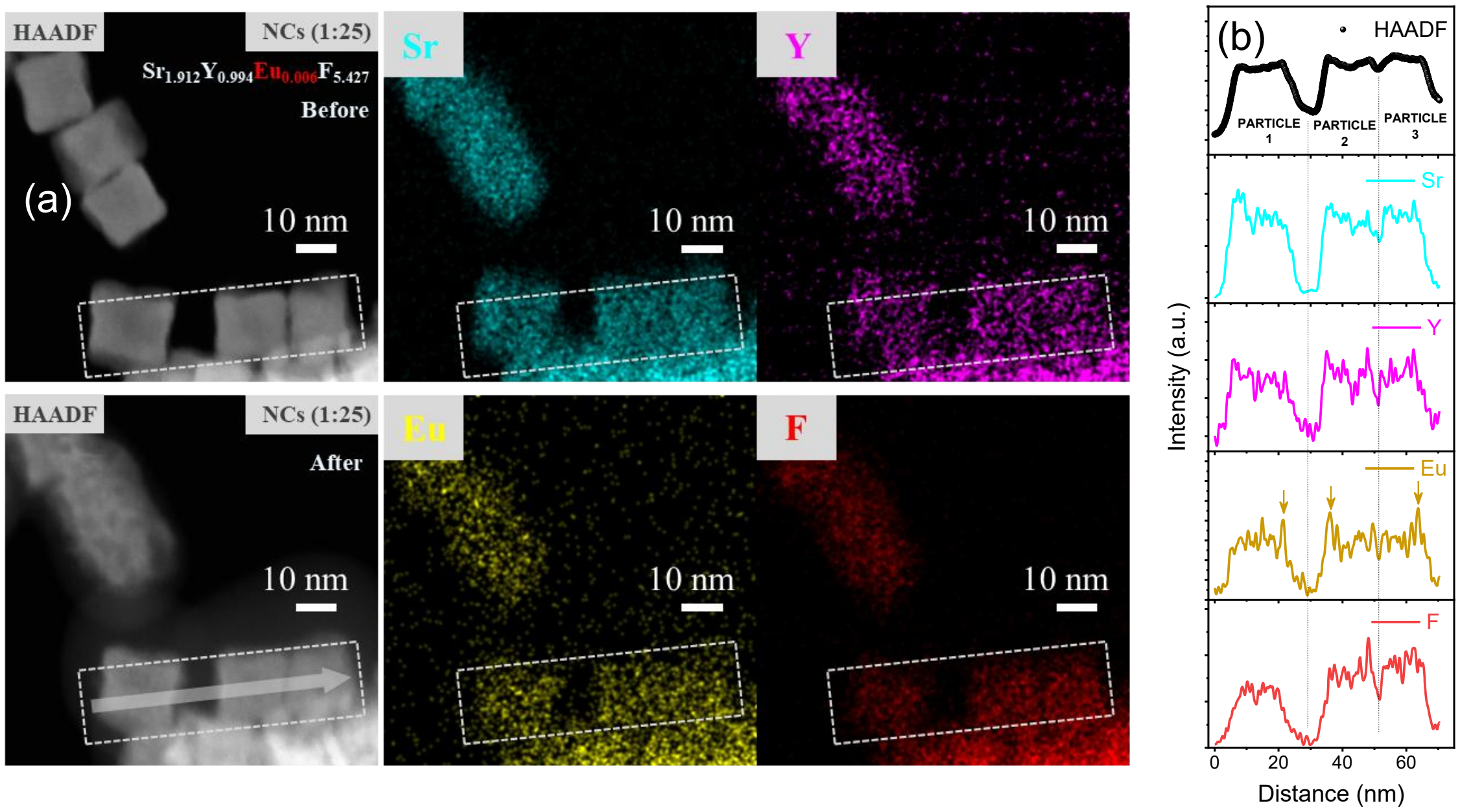

Figure S16. (a) Single particle-level STEM-HAADF image (before and after the measurement) and EDS elemental mapping of Eu^{3+}-exchanged NCs (1:25). The chemical composition of the sample as presented is estimated based on the EDS analysis. (b) HAADF and Sr, Y, Eu, and F elemental profiles of NCs at a single particle level, with the region of signal collection marked as the square in the figure. Some traces of the signal maxima of Eu element at the edge region of a single NC are marked by arrows.

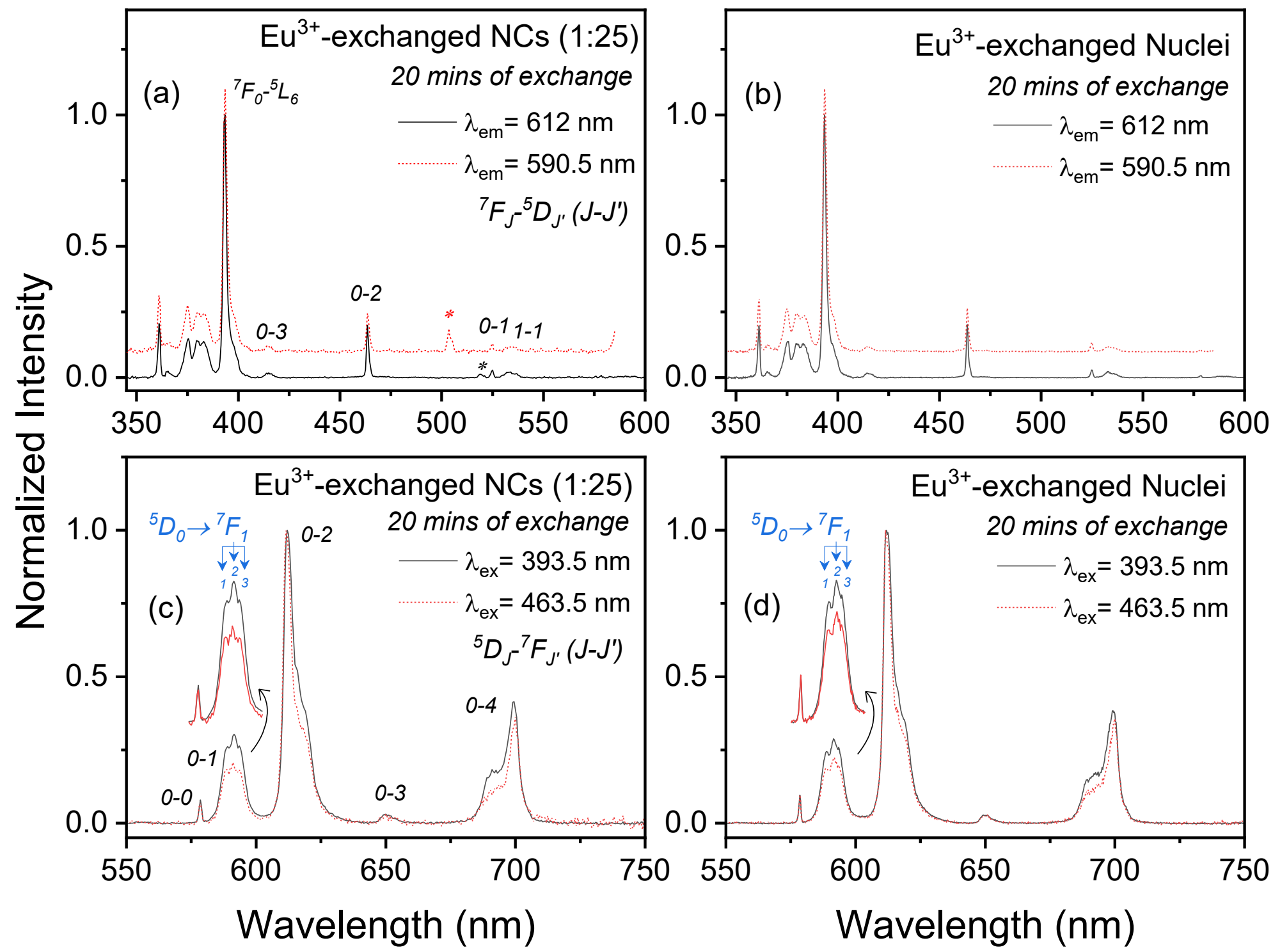

Figure S17. (a, b) Normalized excitation and (c, d) emission spectra of the Eu^{3+}-exchanged (20 mins) SYF NCs (1:25) and nuclei by monitoring different emissions and with different excitations, respectively. The terms of the relevant 4*f*–4*f* transitions of Eu^{3+} are indicated. The insets (c, d) give the enlargement of the corresponding emission spectra of samples, showing a three-fold splitting of the Eu^{3+} $^5D_0$–$^7F_1$ transition in all cases, despite the noticeable difference in their relative intensities, with a sole $^5D_0$–$^7F_0$ line of Eu^{3+} collected.

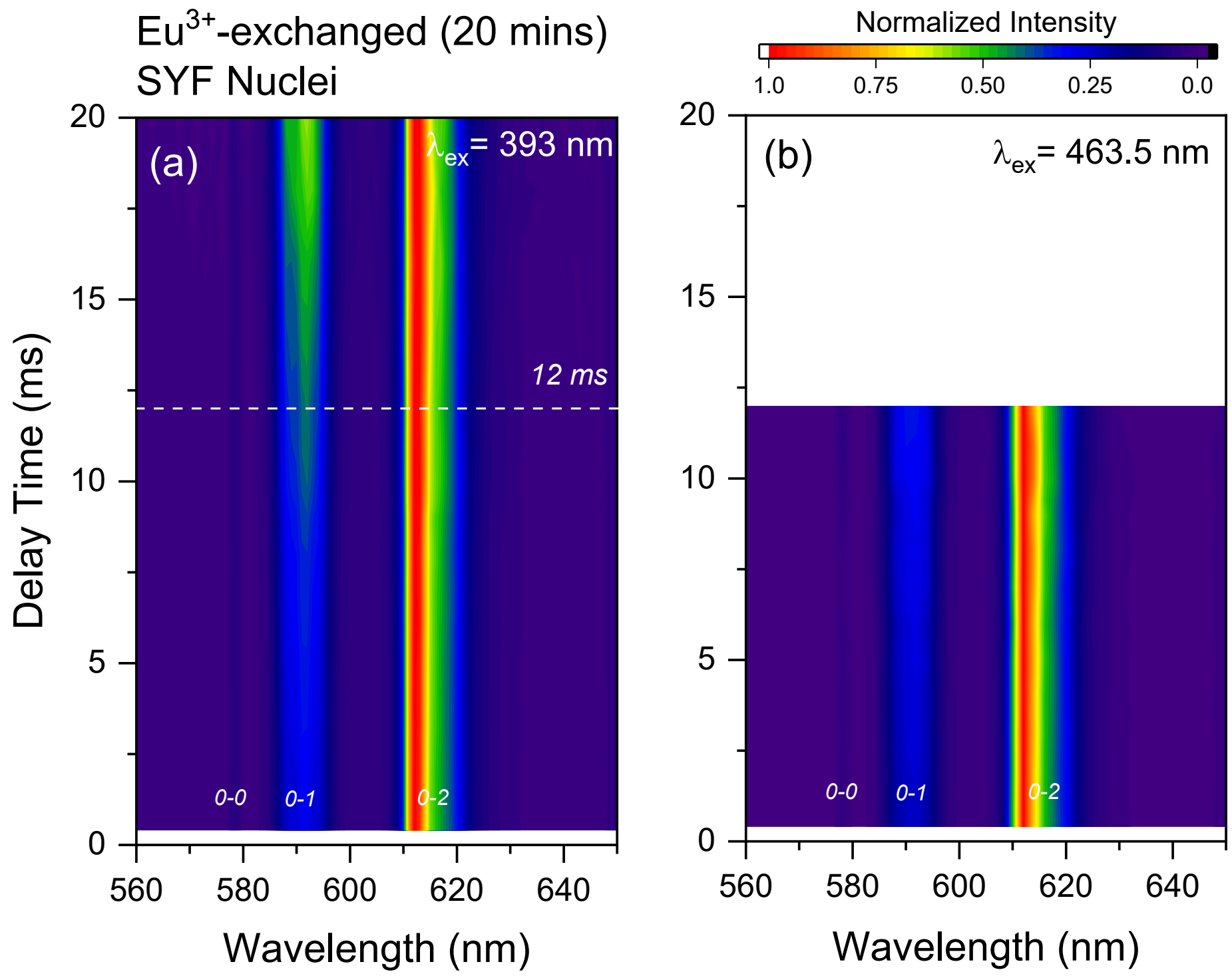

Figure S18. Color contours of the normalized time-resolved emission spectra (TRES) of the Eu^{3+}-exchanged (20 mins) SYF nuclei upon excitations of (a) the Eu^{3+} $^7F_0$–$^5L_6$ (393 nm) and (b) $^7F_0$–$^5D_2$ transitions (463.5 nm), respectively.

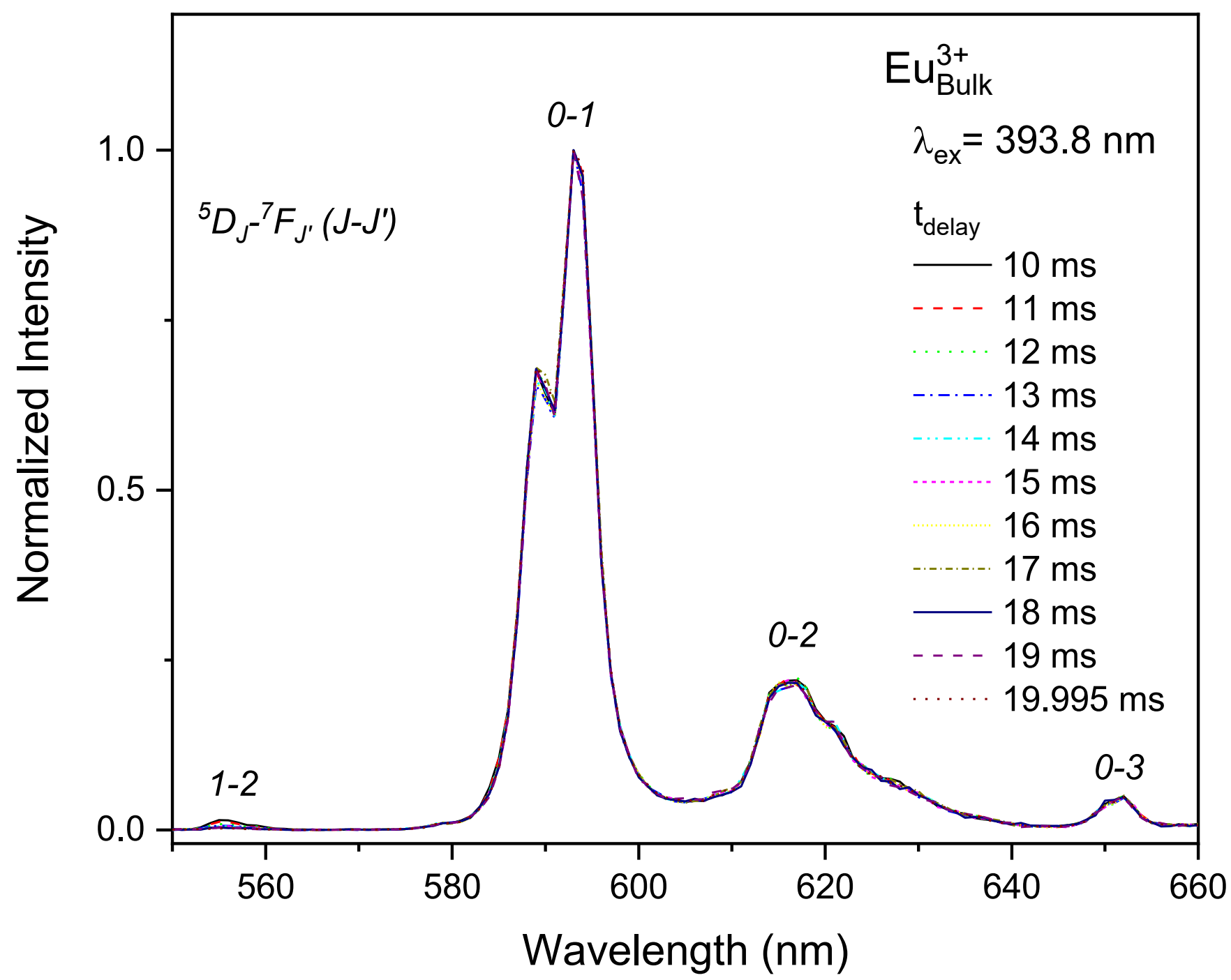

Figure S19. Normalized TRES of the bulk Eu^{3+} residing at Wyckoff 4*a* cation site in the lattice of SYF upon excitation of the Eu^{3+} $^7F_0$–$^5L_6$ (393.8 nm) transition with different signal acquisition delays.

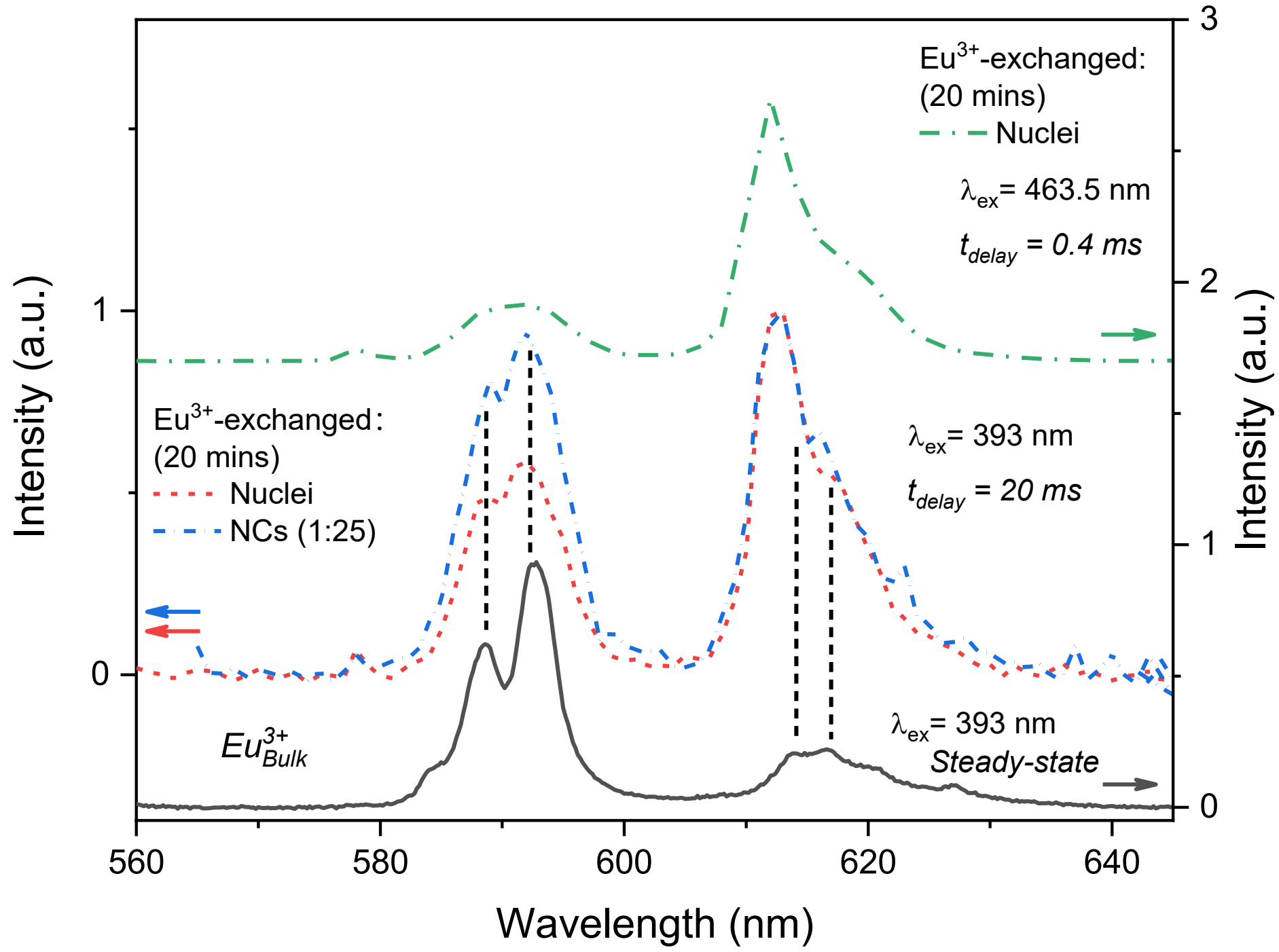

Figure S20. Comparison of the normalized emission spectra of Eu^{3+} acquired from the TRES slices of the Eu^{3+}-exchanged (20 mins) nuclei and NCs (1:25) with different signal acquisition delays, as well as the steady-state emission spectrum of bulk Eu^{3+}.

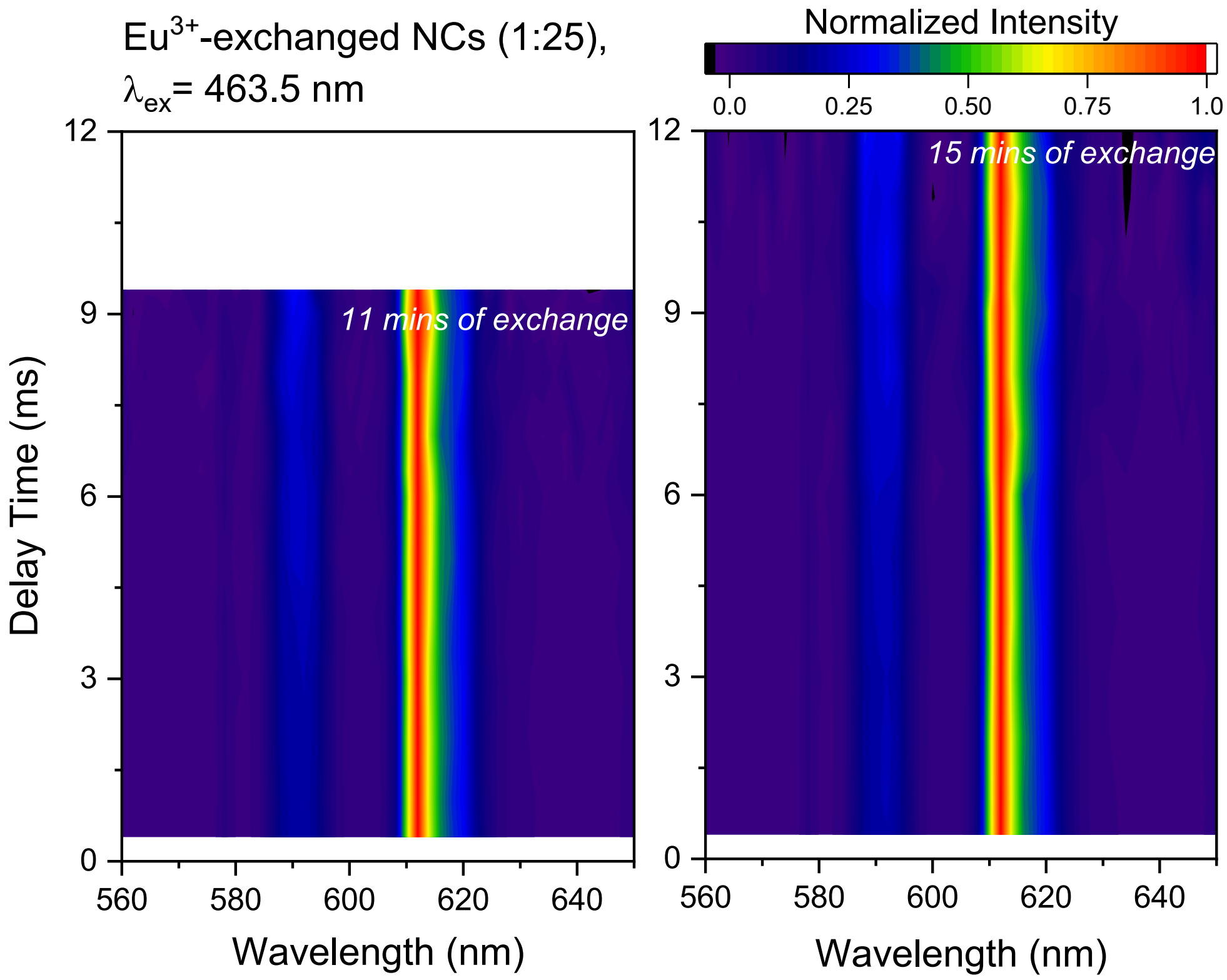

Figure S21. Color contours of the normalized TRES of the Eu^{3+}-exchanged SYF NCs (1:25) with different exchange durations (11 and 15 mins) upon 463.5 nm excitation, respectively.

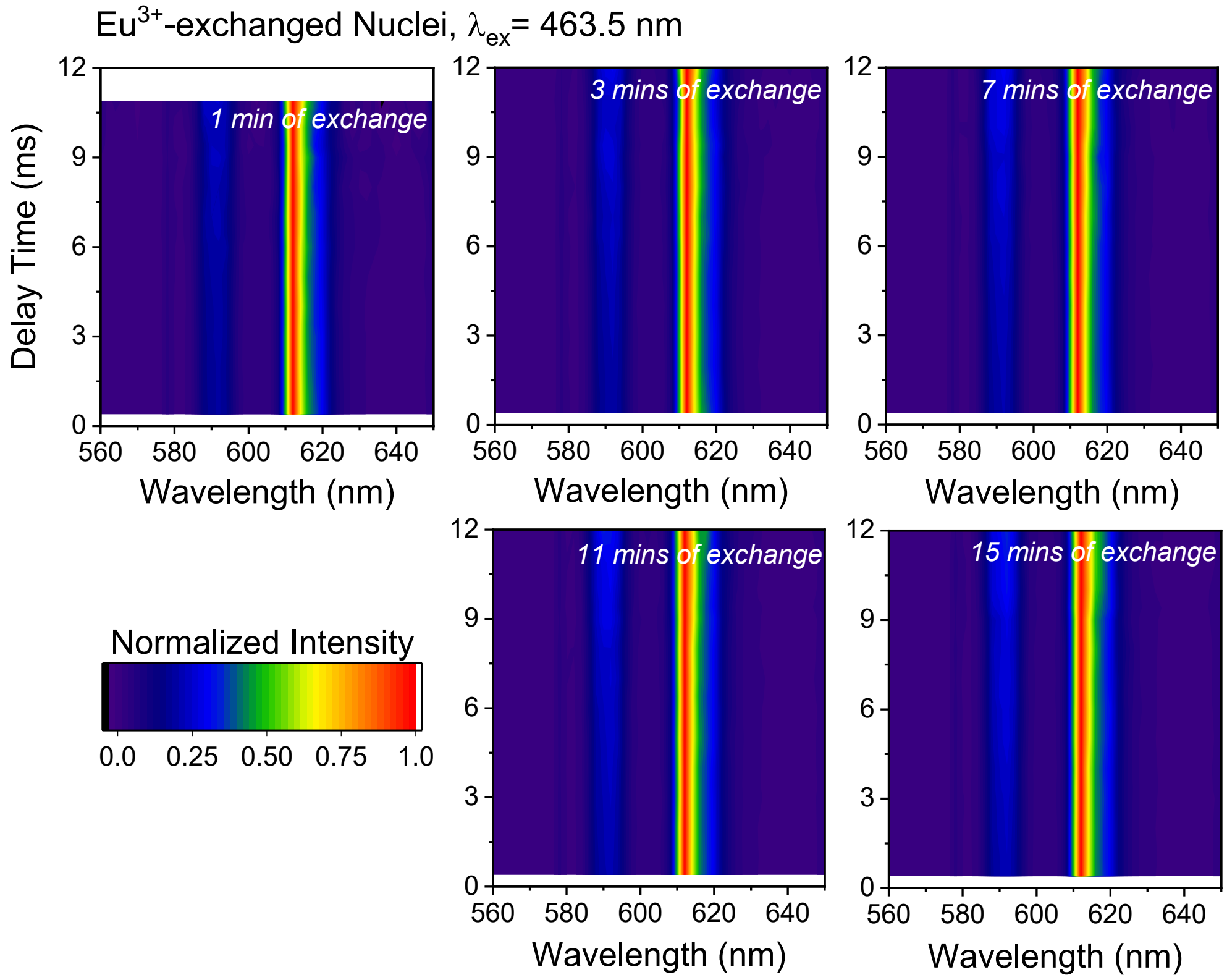

Figure S22. Color contours of the normalized TRES of the Eu^{3+}-exchanged SYF nuclei with different exchange durations (from 1 to 15 mins) upon 463.5 nm excitation, respectively.

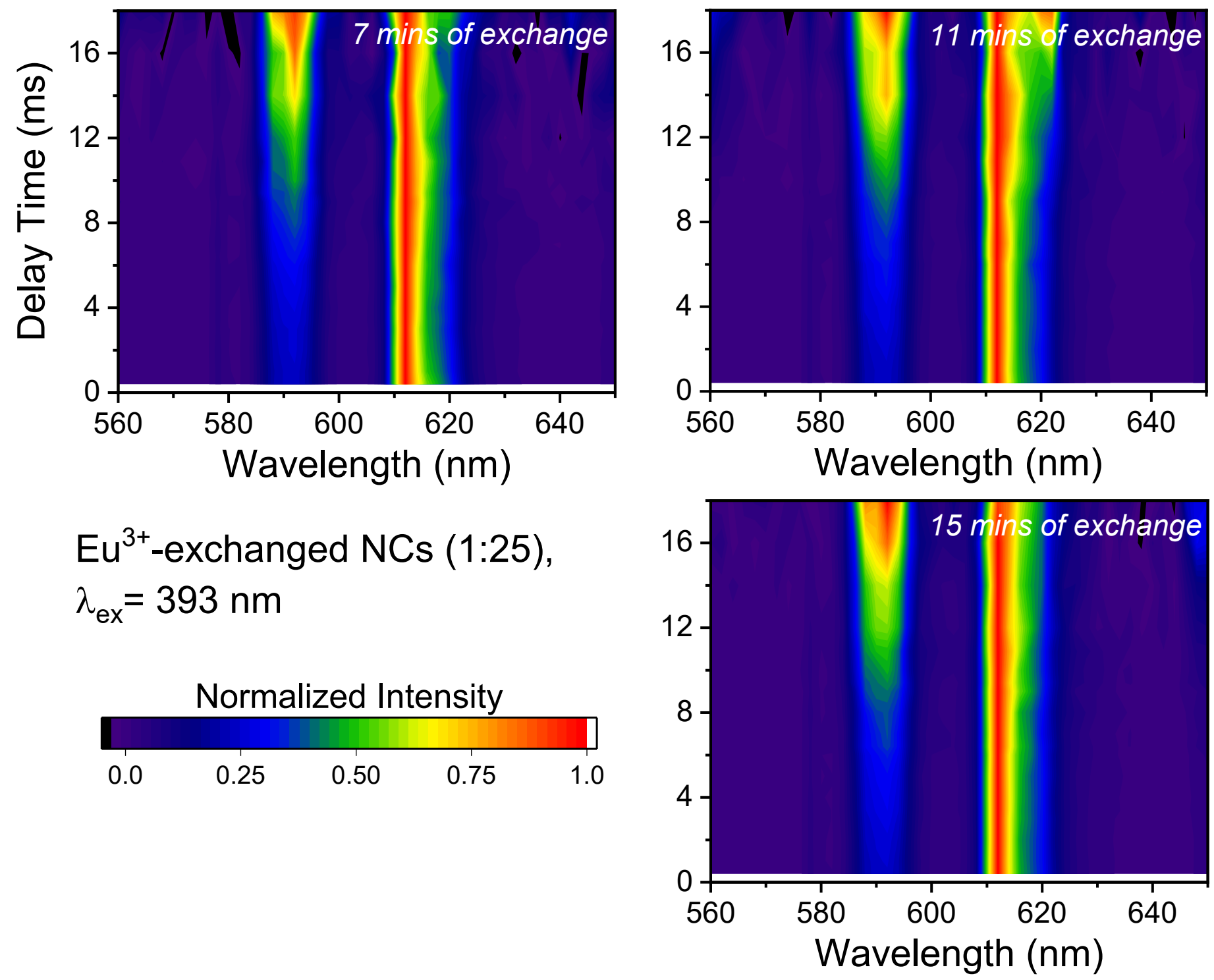

Figure S23. Color contours of the normalized TRES of the Eu^{3+}-exchanged SYF NCs (1:25) with different exchange durations (from 7 to 15 mins) upon 393 nm excitation, respectively.

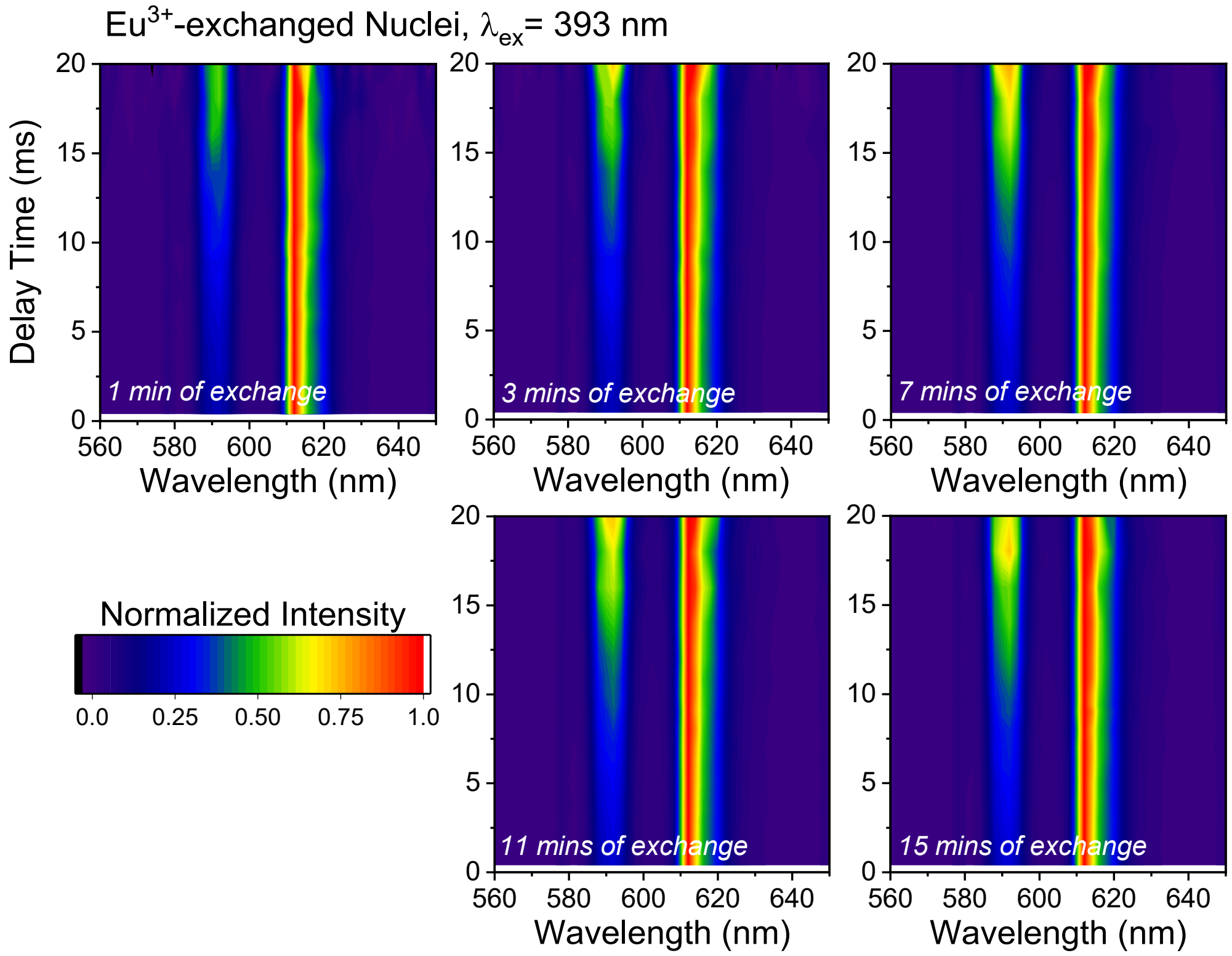

Figure S24. Color contours of the normalized TRES of the Eu^{3+}-exchanged SYF nuclei with different exchange durations (from 1 to 15 mins) upon 393 nm excitation, respectively.

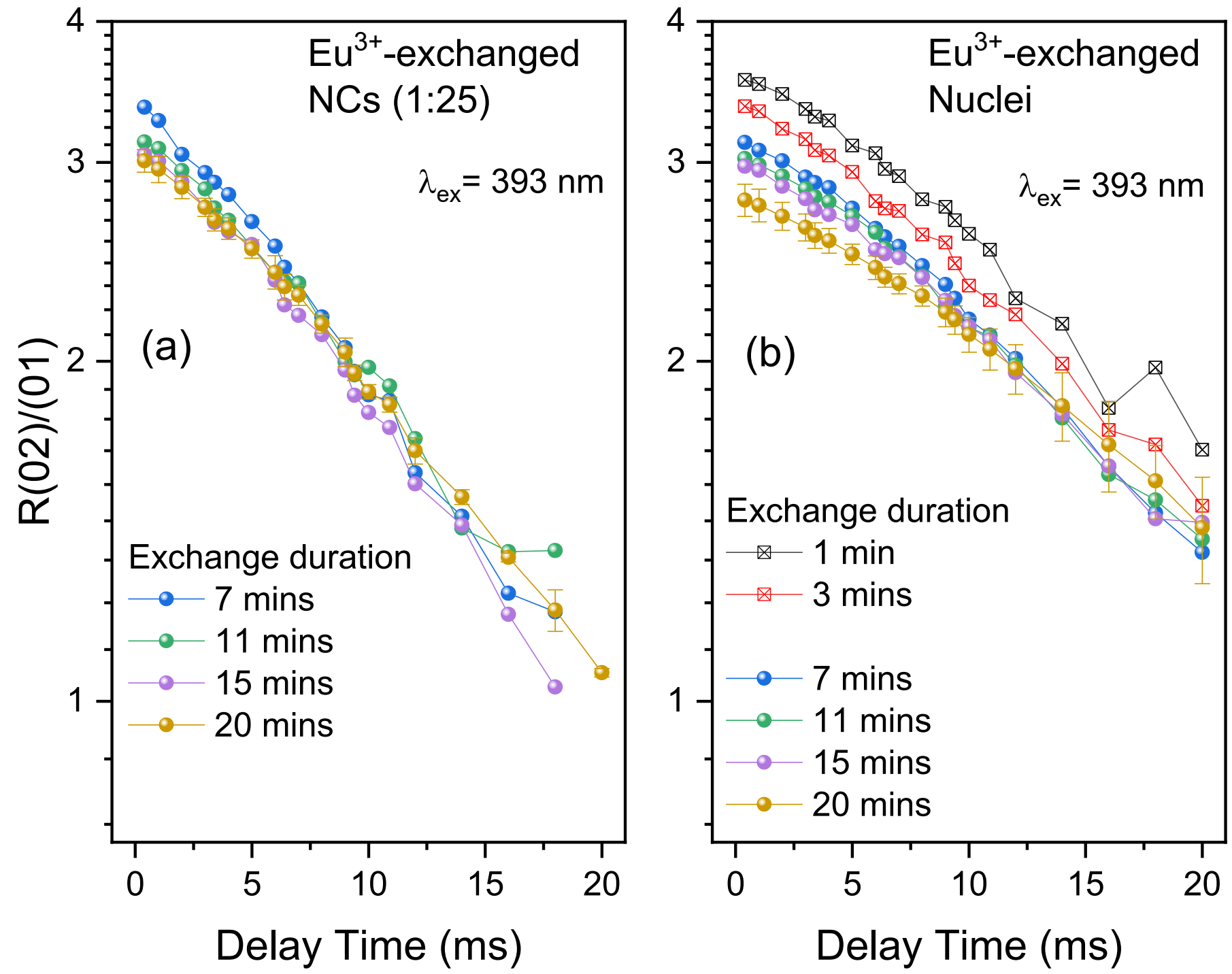

Figure S25. (a) Dependences of the R value of Eu^{3+} emission profile on the signal acquisition delay in the TRES of the Eu^{3+}-exchanged NCs (1:25) and (b) nuclei with different exchange durations upon 393 nm excitation, respectively.

1. Sahoo, Y.; Pizem, H.; Fried, T.; Golodnitsky, D.; Burstein, L.; Sukenik, C. N.; Markovich, G., Alkyl Phosphonate/Phosphate Coating on Magnetite Nanoparticles: A Comparison with Fatty Acids. *Langmuir* **2001,** *17*, 7907-7911.
